# Protective anti-tumor vaccination against glioblastoma expressing the MHC class II transactivator CIITA

**DOI:** 10.3389/fimmu.2023.1133177

**Published:** 2023-03-13

**Authors:** Fabrizio Celesti, Andrea Gatta, Mariam Shallak, Anna Maria Chiaravalli, Michele Cerati, Fausto Sessa, Roberto S. Accolla, Greta Forlani

**Affiliations:** ^1^ Laboratories of General Phatology and Immunology “Giovanna Tosi”, Department of Medicine and Surgery, University of Insubria, Varese, Italy; ^2^ Unit of Pathology, ASST Sette-Laghi, Varese, Italy; ^3^ Unit of Pathology, Department of Medicine and Surgery, ASST Sette-Laghi, University of Insubria, Varese, Italy

**Keywords:** CIITA, tumor vaccination, T helper, MHC-II, glioblastoma

## Abstract

Glioblastoma is the most malignant tumor of the central nervous system. Current treatments based on surgery, chemotherapy, and radiotherapy, and more recently on selected immunological approaches, unfortunately produce dismal outcomes, and less than 2% of patients survive after 5 years. Thus, there is an urgent need for new therapeutic strategies. Here, we report unprecedented positive results in terms of protection from glioblastoma growth in an animal experimental system after vaccination with glioblastoma GL261 cells stably expressing the MHC class II transactivator CIITA. Mice injected with GL261-CIITA express *de novo* MHC class II molecules and reject or strongly retard tumor growth as a consequence of rapid infiltration with CD4+ and CD8+ T cells. Importantly, mice vaccinated with GL261-CIITA cells by injection in the right brain hemisphere strongly reject parental GL261 tumors injected in the opposite brain hemisphere, indicating not only the acquisition of anti-tumor immune memory but also the capacity of immune T cells to migrate within the brain, overcoming the blood–brain barrier. GL261-CIITA cells are a potent anti-glioblastoma vaccine, stimulating a protective adaptive anti-tumor immune response *in vivo* as a consequence of CIITA-driven MHC class II expression and consequent acquisition of surrogate antigen-presenting function toward tumor-specific CD4+ Th cells. This unprecedented approach for glioblastoma demonstrates the feasibility of novel immunotherapeutic strategies for potential application in the clinical setting.

## Introduction

Glioblastoma (GBM), the most malignant tumor of the central nervous system (CNS), accounts for approximately 60% to 70% of all malignant gliomas. Worldwide annual incidence is approximately 0.59 to 5 per 100,000, with a trend toward increasing, particularly in South America and Eastern and Southern Europe (1). GBM has high morbidity and mortality rates. Even with best-to-date treatment, median survival is only 12–15 months (2). The particularly disproportionate mortality is reflected in the fact that although GBM accounts for only 1.4% of all cancers, it represents 2.9% of cancer-related deaths (3). Thus, there is an urgent need for better treatments for this deadly form of cancer.

Additional difficulties in treating GBM and CNS tumors in general reside in the fact that blood–brain barrier (BBB) limits access by therapeutic agents, although this limitation is reduced during brain pathologies, including cancer (4, 5). Due to the existence of the BBB and the idea that it lacked a lymphatic drainage system, the CNS was long believed to be a privileged tissue with respect to immune attack. However, the detection of lymphocytes and tumor cell migration from the brain to cervical lymph nodes has suggested that passage into the lymphatic system could exist (6). Indeed, the existence of a lymphatic system along the draining cerebral sinuses in mice has been demonstrated (7, 8). Moreover, recent reports have shown that there is a local source of immune cells resident in the bone marrow of the skull (9), respond to signals from the environment in the presence of a pathogen or in response to an injury by proliferating and migrating to the site of infection or injury (10).

Taken together, these observations suggest that not only can the immune system reach the CNS, but it is also in close contact with it physiologically. These considerations have revitalized the idea of an immune approach to glioblastomas, and several cell-based treatment modalities, alone or in combination with classical radiotherapy and temozolomide, are now under scrutiny for the treatment of this type of tumor (11–13). Among the possible immunological approaches, attempts have been made to use peptide vaccines targeted mainly at the stimulation of tumor-specific MHC class I-restricted CD8+ cytotoxic T lymphocytes (CTLs), which are important terminal effectors of anti-tumor immunity (14). Less attention has been focused, in contrast, on CD4+ T helper (Th) cells, the critical lymphocyte subpopulation of adaptive immunity (15). Without triggering of Th cells, effector CTLs cannot proliferate and their presence cannot be maintained for a long time, particularly *in vivo* (16). Th cells are triggered by peptide antigens presented by MHC class II (MHC-II) molecules on the surface of classical antigen-presenting cells (APCs), including dendritic cells (DCs), macrophages, and B cells (17, 18). Based on this evidence, our approach to stimulate the complex mechanism of anti-tumor immunity involves the modification of the tumor cells through genetic transfer of the MHC class II transactivator, also designated CIITA (19), the crucial physiological activator of MHC class II gene expression discovered in our laboratory (20–22). Most tumor cells do not express MHC class II molecules because of a lack of expression of CIITA. Once transformed with CIITA, tumor cells express MHC class II molecules and thus may act as surrogate APCs for their own tumor antigens for optimal presentation to tumor-specific Th cells (23, 24). In a large series of studies, we have been able to validate this hypothesis in many tumor experimental models of distinct histological origin, including carcinomas and sarcomas (23, 25–27).

Here, we describe for the first time the application of our tumor vaccination strategy to glioblastoma, a tumor that does not express CIITA (28) in a constitutive fashion but can be induced to do so after stimulation with IFN-γ, at least *in vitro* (28, 29). We show that genetic transfer of CIITA into the murine glioblastoma model cell line GL261 renders these cells MHC class II-positive and potent stimulators of an adaptive immune response *in vivo* when injected intracranially. This response protects the mouse from tumor take or strongly retards the tumor growth. Protected animals develop an anti-tumor anamnestic response capable of rejecting or strongly counteracting subsequent challenges with parental tumor cells. Importantly, accurate analysis of the tumor tissue demonstrates that tumor rejection correlates with increased infiltration of T lymphocytes, particularly CD4+ and CD8+ T cells, but not B cells or natural killer (NK) cells, and with profound subversion of the tumor microenvironment.

These results represent the first evidence that a protective adaptive immune response against the most deadly tumor of the CNS may be generated *in vivo* by inducing the tumor cells to express CIITA and thus their own MHC class II molecules that may serve, as demonstrated previously in other tumor models, to present their own tumor antigens.

## Materials and methods

### Generation of GL261 cells stably expressing CIITA

The Glioma 261 (GL261) cell line (30, 31) was cultured in Dulbecco’s Modified Eagle Medium (DMEM) (Lonza BioWhittaker™, Durham, NC, USA) supplemented with 10% heat-inactivated fetal calf serum (FCS). GL261 tumor cells were transfected with 5 μg of flag-CIITA (pc-fCIITA) expression vector (32) using FugeneHD (Promega, Madison, WI, USA), as previously described (33).

Transfected GL261-CIITA cells underwent selection in a medium containing 0.5 mg/ml G418 (Sigma Chemical Corp., St. Louis, MO, USA). MHC-II-positive cells were enriched by fluorescence-activated cell sorting with a BD FACS ARIA II cell sorter (Becton-Dickinson, Franklin Lakes, NJ, USA) and further subjected to limiting dilution cloning. In detail, GL261-CIITA cells sorted for MHC-II-positive expression were diluted to 5–10 cells/ml, and 100 μl/well was dispensed into two 96-well plates. At least 50 clones were analyzed and further selected for high MHC-II cell surface expression.

### Measurement of *in vitro* growth rate

GL261 or GL261-CIITA cells (5 × 10^4^ cells per well) were seeded in 48-well plates, and cell proliferation was measured at 24, 48, and 72 h by counting the cells using trypan blue exclusion assay. Each point in the growth curve was obtained from three independent experiments performed in triplicate wells.

### Fluorescence-activated cell sorting analysis

Cell surface expression of both MHC-I and MHC-II molecules was assessed by immunofluorescence and flow cytometry (BD FACSAria™ II Cell Sorter, BD Biosciences, San Jose, CA, USA) using an anti-H-2 K/D class I monoclonal antibody (clone M1/42, BioLegend, San Diego, CA, USA) and anti-IA/IE class II monoclonal antibody (clone M5/114.15.2, BioLegend), respectively. Negative controls were obtained by staining the cells with specific isotype-matched antibodies.

### Western blotting

Cell lysates obtained from either GL261 or GL261-CIITA cells (4 × 10^6^ cells) were analyzed for expression of CIITA using sodium dodecyl sulfate–polyacrylamide gel electrophoresis (SDS-PAGE) and Western blotting with the anti-CIITA 71H (Santa Cruz Biotechnology, Dallas, TX, USA) monoclonal antibody. The expression of α-tubulin was assessed with an anti-α-tubulin monoclonal antibody (Sigma). Horseradish peroxidase-conjugated anti-mouse Ig secondary antibody was used (Thermo Scientific, Waltham, MA, USA). Blots were developed by chemiluminescence assay (SuperSignal West Pico; Thermo Scientific).

### Animal models

C57BL/6 (H-2^b^ genotype) mice aged 7–8 weeks were purchased from Charles River (Charles River Laboratories Italia SRL, Calco, Italy). Each experiment was repeated at least twice using five to eight mice per group.

All animal experiments were conducted according to relevant national and international guidelines and were approved by the University of Insubria Internal Ethics Committee CESA and by the Italian Ministry of Health (Project 05-2020).

### Intracranial tumor injection

All surgical procedures were conducted within the animal housing room. All mice were anesthetized with ketamine (100 mg/kg) and xylazine (15 mg/kg) administered by intraperitoneal injection, preceded by subcutaneous tramadol injection (5 mg/kg). Surgical anesthesia was confirmed by the loss of the pedal reflex. Mice were positioned in a stereotactic frame; the skull was cleaned with a sterile cotton swab soaked in 70% ethanol, and a 5–7-mm sagittal incision was made through the scalp. A small hole was drilled 1.5 mm posterior and 2 mm lateral (right) to the bregma.

A total of 3 × 10^4^ GL261 or GL261-CIITA cells in 3 μl of serum-free medium were injected 3 mm deep from the dura intracranially using a 26-gauge needle. Mice were monitored daily for signs of brain tumor growth, such as seizures, ataxia, or weight loss, and were sacrificed before the planned day of the experiment if the tumor burden became symptomatic. Three weeks after injection of the tumor cells, mice were sacrificed, and their brains were harvested.

In tumor challenge experiments, mice previously injected with 3 × 10^4^ GL261-CIITA cells into the right striatum were injected in the left striatum with 3 × 10^4^ GL261 parental tumor cells 3 weeks post-injection. As a control, 3 × 10^4^ GL261 parental tumor cells were injected into the left striatum of non-vaccinated mice. All mice were sacrificed after an additional 3 weeks, and their brains were prepared for histology analysis as described below.

### Immunohistochemistry

All mouse brains were completely sampled, formalin-fixed, and paraffin-embedded. From each paraffin block, serial sections of 3-µm thickness were cut, mounted on positively charged slides, and stained in hematoxylin and eosin (HE) for morphological evaluation or use in the immunohistochemical analysis, as follows. Brain sections were deparaffinized, rehydrated, and treated with hydrogen peroxide 3% solution for 20 min to inhibit endogenous peroxidases. After they had been washed in TBS with 0.25% triton X-100 (Sigma Chemical Corp., St. Louis, MO, USA), antigen retrieval was performed in a microwave oven at 700-W power for the specified time (**Table 1**), using citrate buffer pH 6 or EDTA buffer pH 8 based on the experimental protocols detailed in **Table 1**. The tissue sections were then incubated overnight at 4°C with the specific primary antibody at the working dilution detailed in **Table 1**. On the following day, the tissue sections were washed in TBS with 0.25% triton X-100 and incubated for 45 min at room temperature (RT) with the specific biotinylated secondary antibody (Vector, Newark, CA, USA) and, subsequently, for 30 min at RT with ABC peroxidase complex (ABC Elite, Vector, Newark, CA, USA). The immunoreaction was developed with 3,3′-diaminobenzidine tetrahydrochloride (DAB) (Sigma Aldrich, St. Louis, MO, USA) as a chromogen. After nuclear hematoxylin counterstaining, the tissue sections were dehydrated through an alcohol scale and mounted with a cover slide using Canada balm. The immunostained brain tissues were analyzed under a light microscope (Olympus, Tokyo, Japan).

**Table 1:** Antibody reagents used in this study.

Antibody specificity	Source	(Clone)^a^	Antigen retrieval^b^	Working dilution
CD3	Thermo Fisher (MA5-14524)	Rb (*SP7*)	E (20 min)	1/150
CD4	Abcam (Ab183685)	Rb (*EPR19514*)	C (20 min)	1/1,000
CD8	Abcam (Ab209775)	Rb (*EPR20305*)	E (20 min)	1/1000
CD11b	Abcam (Ab133357)	Rb (*EPR1344*)	E (10 min)	1/20,000
CD11c	Abcam (Ab219799)	Rb (*EPR21826*)	E (10 min)	1/400
CD19	Thermo Fisher (14-0194-80)	Rat (*6OMP31*)	E (20 min)	1/1,000
CD161	Abcam (Ab234107)	Rb (*EPR21236*)	C (20 min)	1/10,000
FoxP3	Thermo Fisher (14-5773-82)	Rat (*FJK-165*)	C (20 min)	1/100
GFAP	Genetex (GTX108711)	Rb	C (20 min)	1/2,000
Ki67	Abcam (Ab16667)	Rb (*SP6*)	C (20 min)	1/100
MHC-II	Thermo Fisher (14-5321-82)	Rat (*M5/114.15.2*)	E (10 min)	1/100
Nestin	Abcam (Ab221660)	Rb (*EPR22023*)	C (20 min)	1/4,000
Synaptophysin	Abcam (Ab32127)	Rb (*YE269*)	C (20 min)	1/6,000

a Rb, rabbit.

b E, EDTA buffer pH 8.0; C, citrate buffer pH 6.0; times in parentheses, incubation time.

### Analysis of tumor size and immune infiltrating cells

Tumor size was defined as the largest area occupied by tumor cells, measured on all consecutive sections stained in the process of both HE and immunohistochemistry using a computer image analysis system (Olympus Software cell Sens Entry Version 4.1). Immune infiltration was evaluated by counting the positive cells in 10 consecutive images taken at ×200 magnification (0.28 mm^2^/field) across the neoplasia, beginning from the edge of tumor growth, including the peritumoral tissue, along the orthogonal axis. In small tumors, when it was not possible to measure 10 tumor fields, immunoreactive cells were counted over the entire surface of the tumor. The value was expressed as the number of positive cells per square millimeter (34). The edge of tumor growth, defined as the invasive margin (IM), is the border separating the host tissue from the malignant nests. Peritumoral tissue is defined as the region outside the IM when it is centered in a ×200 field (500-µm extension) (35) (**Supplementary Figure 1**). In the absence of tumor cells, the lymphocyte cell count was performed throughout the tumor bed (TB) area and extending from the TB edge over a 500-µm radius. The TB is defined as the tissue encompassing the original tumor site (36); microscopically, the TB is characterized by an area of hyalinized, edematous reactive stroma with a plethora of inflammatory infiltrating cells (36–38).

### Statistical analysis

Statistical analysis was performed using GraphPad Prism 6 (GraphPad Software, http://www.graphpad.com), and the Student’s t-test was conducted to determine significance. Comparisons were considered statistically significant when the corresponding p-value was <0.05. All data are expressed in the form mean ± standard deviation (SD).

## Results

### MHC-II-positive GL261-CIITA tumor cells are rejected or their growth significantly reduced *in vivo* after intracranial implantation

**Figure 1:**
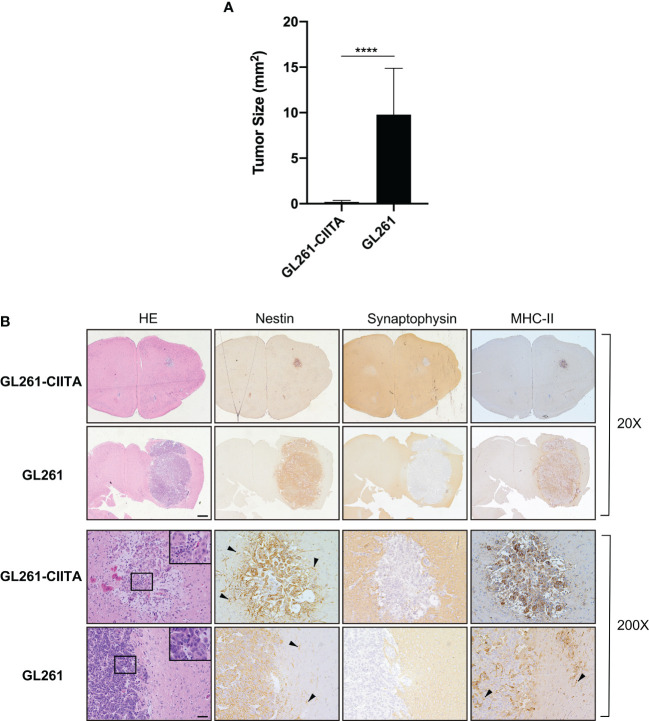
Intracranial implantation of MHC-II-positive GL261-CIITA tumor cells dramatically retarded tumor growth *in vivo.* C57BL/6 mice received intracranial injection of 3 × 10^4^ GL261 (n = 7) or GL261-CIITA (n = 10) glioma cells. On day 21 after injection, mice were sacrificed, brains were removed, and serial sections of the brain were carried out to measure tumor size and for staining. **(A)** Average tumor size of GL261 and GL261-CIITA tumors. Data are represented as mean values, and error bars indicate the standard deviation (SD) within each group; n = 10. *p*-Values were determined *via* unpaired t-test; ****p < 0.0001. **(B)** HE and IHC staining of serial brain sections. The first two series of upper panels were taken at ×20 magnification; scale bar corresponds to 500 μM. The third and fourth series of horizontal panels were taken at ×200 magnification; scale bar corresponds to 50 μM. Small square boxes are the areas represented in the corresponding large square boxes at ×400 magnification of each IHC image. Note that selected areas in IHC images of GL261 parental tumors are taken as representative of high mitotic cell rate. Arrowheads in the nestin-stained panels point to astrocytes, and in the MHC-II stained panel to myeloid cells. HE, hematoxylin and eosin; IHC, immunohistochemistry.

Upon stable CIITA transfection, the MHC-II-negative GL261 cell lines (GL261) displayed a stable MHC-II IA-positive phenotype, as assessed by flow cytometry and immunofluorescence (**Supplementary Figure 2A**). As expected, CIITA was expressed only in GL261-CIITA cells, as assessed by Western blot (**Supplementary Figure 2B**). To verify whether *de novo* CIITA-driven MHC-II expression in GL261 cells could affect GL261 tumorigenicity, GL261-CIITA or GL261 parental cells were orthotopically implanted in syngeneic C57BL/6 mice. Three weeks after tumor injection, the mice were sacrificed, their brains were harvested, and the tumor mass was analyzed by immunohistology. GL261-CIITA tumors were strongly retarded in their *in vivo* growth with respect to GL261 parental tumor. Importantly, 10% of mice fully rejected GL261-CIITA tumor cells. The average size of parental tumors in GL261-CIITA-injected mice was 50 times smaller than that of tumors in GL261-injected mice (average tumor size: 0.19 versus 9.8 mm^2^; unpaired Student’s t-test, *p* < 0.0001) (**Figure 1A**). HE staining, in conjunction with nestin and synaptophysin staining, was carried out to assess the extent of the tumor mass. Nestin is a neural stem cell marker expressed in glioblastomas and GL261 (39), whereas synaptophysin is a marker of glioneuronal elements and is not expressed in glioblastomas (40).

Representative brain histological sections stained with HE clearly showed that GL261 tumors were characterized by marked cellular atypia and numerous abnormal mitotic figures (**Figure 1B**, inset ×400). In contrast, GL261-CIITA tumors were characterized by solid cordons and nests, and were associated with loose tissue, a peritumoral edematous area, and a low mitotic rate (**Figure 1B**). An abundant inflammatory infiltrate was uniformly spread throughout the tumor mass as well as in the peritumoral area (**Figure 1B**, inset ×400).

Moreover, the evaluation of tumor sections immunostained for nestin and synaptophysin showed that GL261 parental tumors were characterized by a sharp boundary between the neoplastic mass and normal healthy parenchyma, with a more invasive pattern of growth than that observed in GL261-CIITA tumors (**Figure 1B**). Of note, nestin was strongly expressed not only on tumor cells but also on the peritumoral astrocytic population (**Figure 1B**, black arrowheads). MHC-II expression was also assessed and was specifically observed on GL261-CIITA tumor cells in the nucleus as well as in the cytoplasm and cell surface (**Figure 1B**). Weak MHC-II-positive staining was found on myeloid cells of the brains of GL261 tumor-bearing mice, both surrounding the tumor and interspersed within the tumor mass (**Figure 1B**, black arrowheads).

The number of proliferating cells, as assessed by Ki67 staining, was clearly higher in GL261 parental tumors compared to GL261-CIITA tumors (**Supplementary Figure 3**, black arrowheads), consistent with the high number of mitotic figures observed in HE stained sections, although this did not correlate with the *in vitro* growth kinetics of GL261 and GL261-CIITA cells that showed a similar proliferative rate (**Supplementary Figure 4**). Brain sections were also stained for glial fibrillary acidic protein (GFAP) to investigate whether CIITA-driven MHC-II expression could affect the level of expression and activation of the surrounding/infiltrating astrocytes. Interestingly, we observed weak positive GFAP staining within the tumor, both in GL261-CIITA and in parental tumors, while a reactive, dense, and more marked peritumoral astrocytosis was present in GL261-CIITA compared to GL261 parental tumor. In GL261 parental tumors, the astrocytic population appeared to be quantitatively decreased and not completely organized to form a peritumoral lining (**Supplementary Figure 3**).

### Increased intratumoral CD4+ and CD8+ T-cell infiltration in GL261-CIITA-injected mice

**Figure 2:**
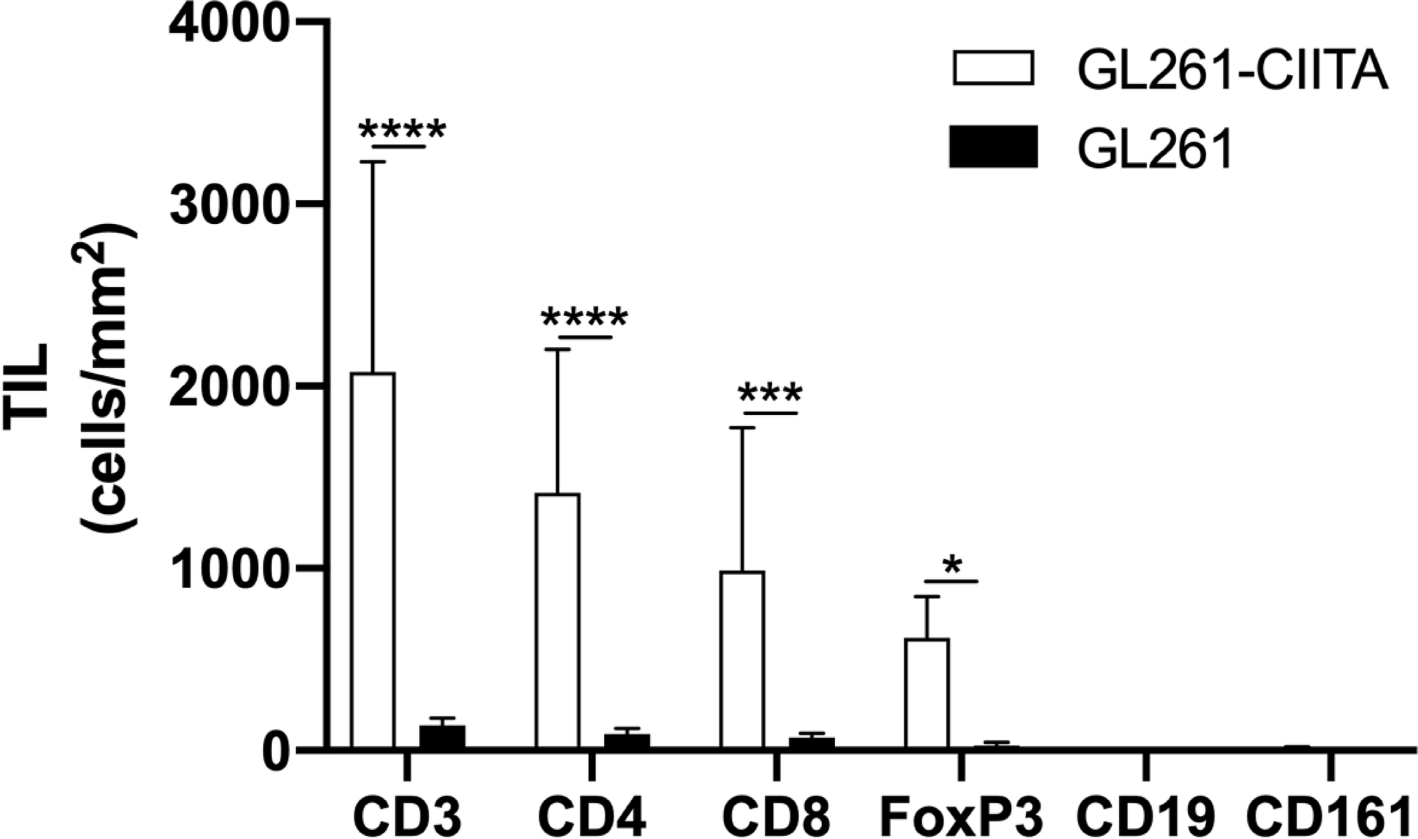
GL261-CIITA tumors are strongly infiltrated by both CD4 and CD8 T cells: quantification. C57BL/6 mice received intracranial injection of 3 × 10^4^ GL261 or GL261-CIITA glioma cells. On day 21 after injection, mice were sacrificed, brains were removed, and serial sections of the brain were carried out for staining with anti-CD3, anti-CD4, anti-CD8, anti-FoxP3, anti-CD19, and anti-CD161 antibodies. Bars represent the average number of CD3, CD4, CD8, FoxP3, CD19, and CD161 tumor-infiltrating lymphocytes (TILs) measured from histopathological sections, as indicated in the Materials and Methods section. Bars represent mean values, and error bars indicate the SD for each group; n = 7. *p*-Values were determined *via* unpaired t test; *CD3:* ****p < 0.0001; CD4: ****p < 0.0001; CD8: ***p < 0.001; FoxP3: *p < 0.05. Neither CD19 nor CD161 was significantly expressed.

**Figure 3:**
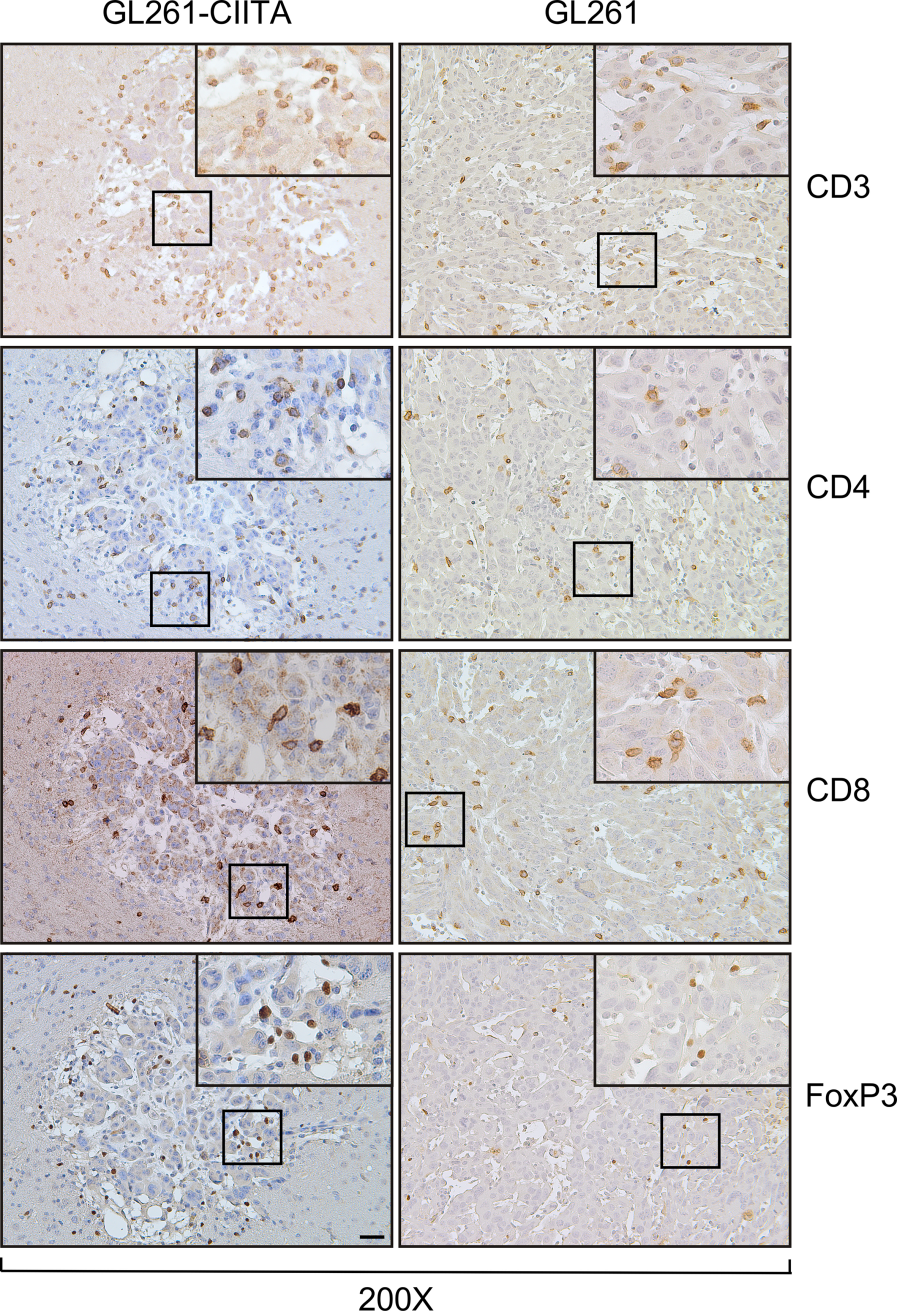
GL261-CIITA tumors are strongly infiltrated by both CD4 and CD8 T cells: immunohistology. C57BL/6 mice received intracranial injection of 3 × 10^4^ GL261 or GL261-CIITA glioma cells. On day 21 after injection, mice were sacrificed, brains were removed, and serial sections of the brain were carried out for staining for CD3, CD4, CD8, and FoxP3 in both GL261 (right panels) and GL261-CIITA (left panels). Small square boxes are the areas represented in the corresponding large square boxes of each IHC image. Images were taken at ×200 magnification; scale bar corresponds to 50 μM. Large square boxes were taken at ×400 magnification. Note that selected areas in IHC images of GL261 parental tumors were taken in the rare zones in which positive cells for the selective marker were present. IHC, immunohistochemistry.

To investigate the possible correlation between inhibition of tumor growth and immune cell infiltrate, the tumor tissue of GL261-CIITA and GL261 was analyzed by immunohistochemistry (IHC) using markers for T cells (CD3, CD4, CD8, and FoxP3), B cells (CD19), and NK cells (CD161). Quantification of tumor-infiltrating cells was performed histologically by systematically screening the entire tumor area from at least three sections obtained from different portions of the tumor, as specified in the Materials and Methods section and in **Supplementary Figure 1**. Tumor infiltrate was very significant in GL261-CIITA tumors as compared to GL261 parental tumors and was mostly represented by CD3+ T lymphocytes, both CD4+ and CD8+ T cells (CD4+: 1,415 cells/mm^2^, CD8+: 989 cells/mm^2^ in GL261-CIITA; CD4+: 138 cells/mm^2^, CD8+: 72 cells/mm^2^ in GL261). Interestingly, T cells expressing FoxP3, a marker usually associated with a regulatory T cell phenotype (Treg) with inhibitory function on CD4+ T cells (41, 42), were more abundant in GL261-CIITA tumors compared to GL261 parental tumor (FoxP3: 618 cells/mm^2^ in GL261-CIITA; 29 cells/mm^2^ in GL261) (**Figure 2**). Thus, although the total number of putative terminal anti-tumor CD8+ T cell effectors was 13.7-fold higher in GL261-CIITA tumors compared to GL261 parental tumors, the ratio of CD8+ T cells to FoxP3+ T cells was lower in GL261-CIITA tumors (CD8+/FoxP3+: mean = 1.8, SD = 1.5) than in GL261 parental tumors (CD8+/FoxP3+: mean = 3.5, SD = 3.2). Interestingly, in both GL261 and GL261-CIITA tumors, most infiltrating T cells were found inside the tumor (CT) and in the invasive tumor margin (IM), while few T cells were localized in the peri-tumor area (PT). In all these different regions, the number of infiltrating T cells was significantly higher in GL261-CIITA tumors than in parental tumors (**Supplementary Figure 5**). Furthermore, the number of T cells infiltrating the tumor was inversely correlated with tumor size (Pearson correlation coefficient: *r^2^* = −0.68, *p* < 0.001). IHC analysis further showed that T cells infiltrating the GL261-CIITA tumor were present both intratumorally and partially dispersed in the peritumoral region (**Figure 3**, insets ×400). Interestingly, in GL261-CIITA tumor, T cells were mainly concentrated along the tumor margins, while in GL261 parental tumor, the smaller numbers of T cells were homogenously distributed throughout the tumor area (**Figure 3**). Very few CD161-positive NK cells were found to be located peritumorally in either GL261-CIITA or GL261 tumors, while CD19-positive B cells were not detected (**Figure 2** and **Supplementary Figure 6**, insets ×400). We also assessed the presence of CD11b- and CD11c-positive cells, representing mostly monocyte/macrophages and dendritic cells, respectively. CD11b-positive cells were more abundant in GL261-CIITA tumors (55%–60%) compared to GL261 (15%–20%) and mostly localized along the tumor margins, similarly to what we observed for GFAP-positive astrocytes. Conversely, in GL261 parental tumor, CD11b-positive cells were mainly dispersed within the tumor area (**Supplementary Figure 6**, insets ×400).

Concerning CD11c-positive cells, these were few in number and present only in GL261-CIITA tumors (**Supplementary Figure 6**, inset ×400).

### The kinetics of growth of GL261-CIITA tumors is significantly delayed compared to that of GL261 parental tumors

**Figure 4:**
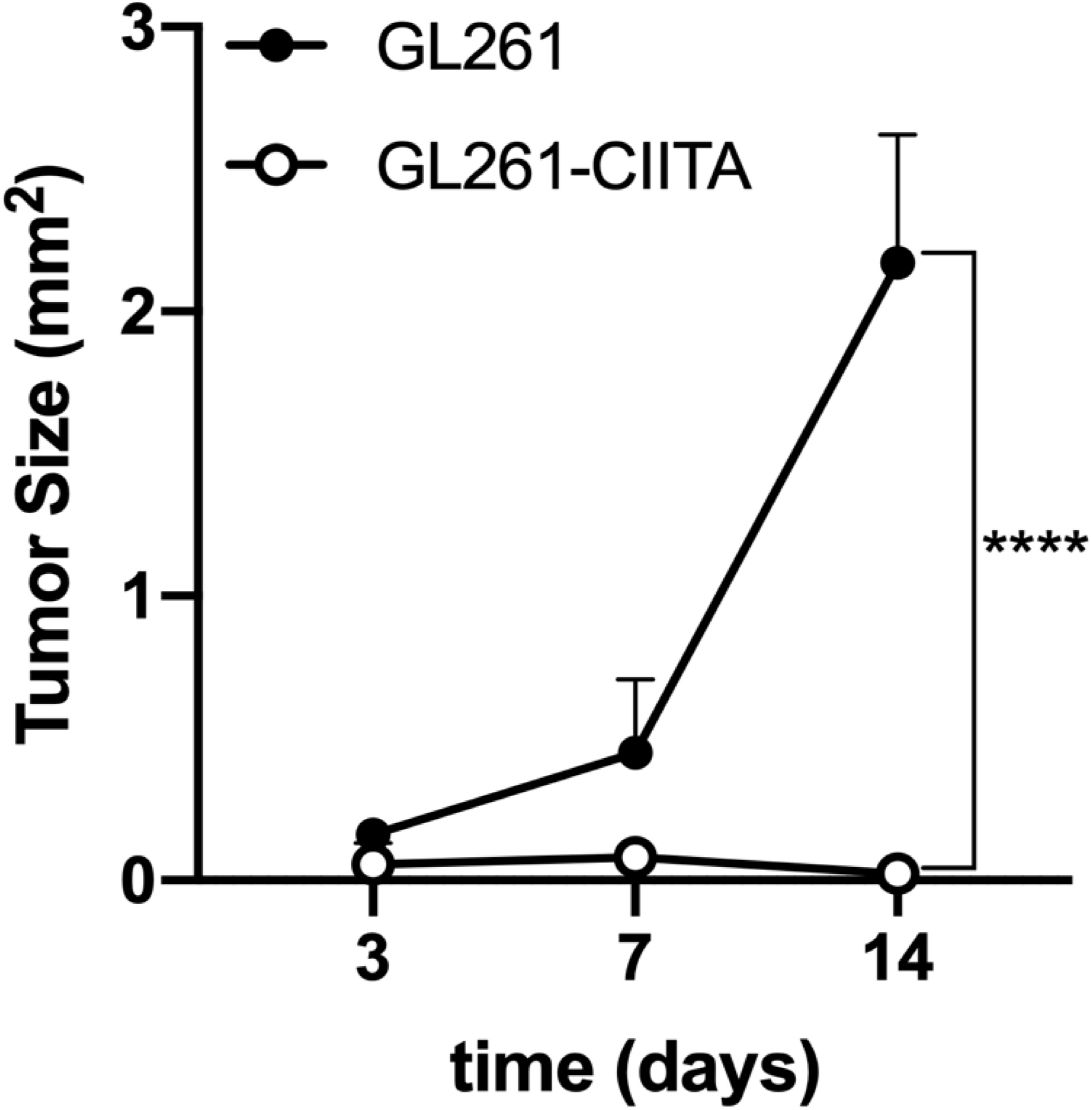
GL261-CIITA tumors are strongly retarded in their growth *in vivo.* C57BL/6 mice received intracranial injection of 3 × 10^4^ GL261 or GL261-CIITA glioma cells. On days 3, 7, and 14 after injection, mice were sacrificed, brains were removed, and tumor size was measured as indicated in the Materials and Methods section. Points represent the average tumor size as mean values, and error points indicate the SD of each group at different time points. *p*-Values were determined *via* unpaired t-test. On day 14, GL261 parental tumors were at least 100 times larger than GL261-CIITA tumors (n = 4, ****p < 0.0001).

As mentioned above and shown in **Supplementary Figure 4**, GL261-CIITA and GL261 parental cells displayed a similar *in vitro* proliferative rate. To investigate the *in vivo* growth kinetics of the GL261-CIITA tumors and compare it to that of the GL261 parental tumors, we monitored tumor growth over time. Mice were sacrificed on days 3, 7, and 14 after tumor cell injection, their brains were harvested, and tumor size was measured as above. As shown in **Figure 4**, on day 3, the average size of GL261 parental tumors was at least twice that of GL261-CIITA tumors; they were four to five times larger on day 7, and a dramatic 100 times larger on day 14.

### Preventive GL261-CIITA vaccination in one brain hemisphere blocks or strongly retards parental tumor growth in the opposite brain hemisphere

To further characterize the CIITA-mediated anti-tumoral response, we verified whether vaccination with CIITA-positive tumor cells in one brain hemisphere could prevent the growth of parental tumor cells in the opposite hemisphere. For this purpose, C57BL/6 mice were injected with GL261-CIITA cells into the right striatum and challenged after 21 days with parental GL261 tumor cells in the left striatum (pre-vaccinated mice). After three additional weeks, the animals were sacrificed, and their brains were analyzed for the presence and size of tumors, as specified above. As a control, at day 21, another group of mice were intracranially injected with GL261 cells, and their brains were analyzed after 3 weeks as above (non-vaccinated).

**Figure 5:**
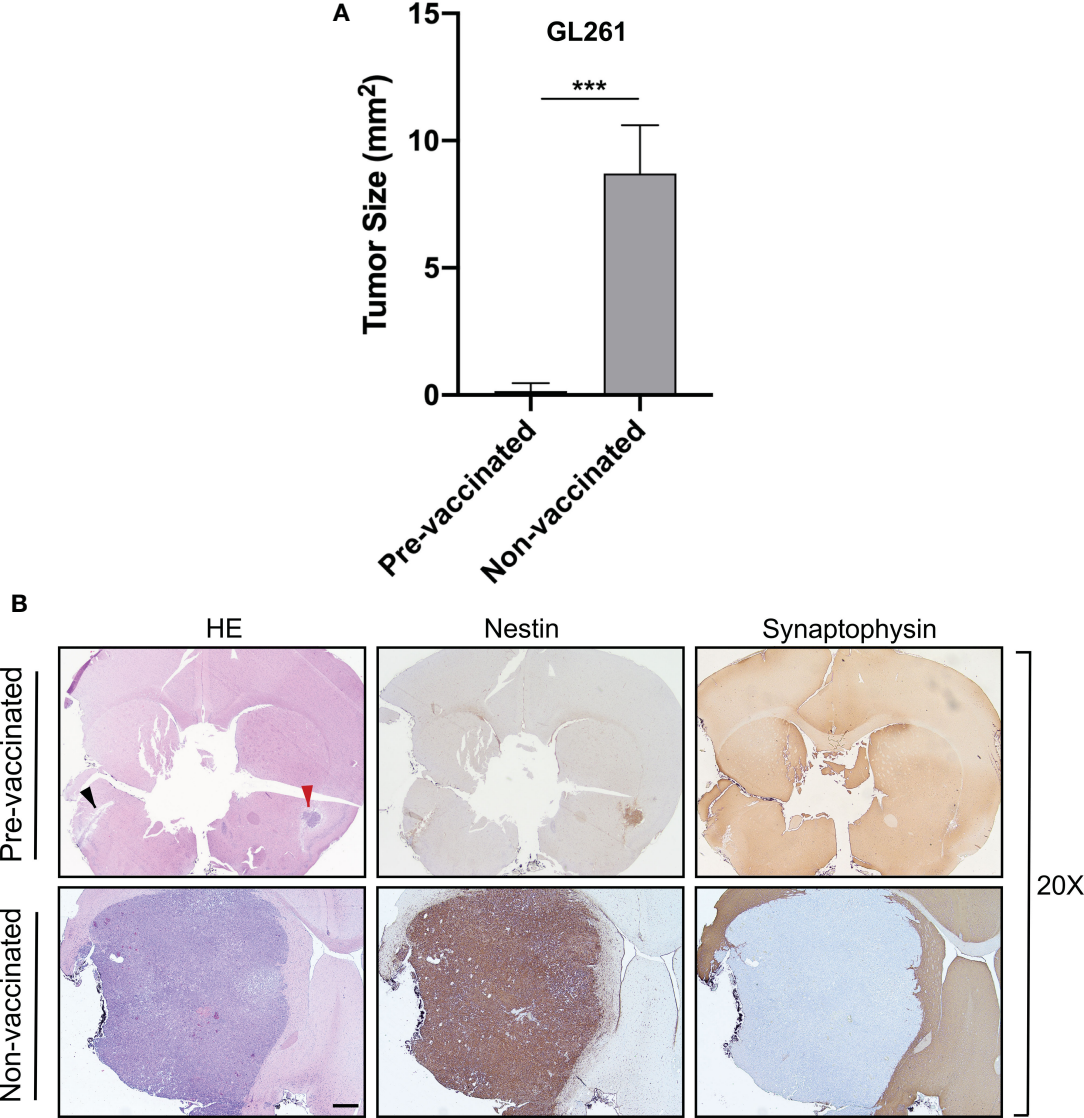
Preventive vaccination with GL261-CIITA tumor cells protects the animal against challenge with GL261 parental tumor cells. C57BL/6 mice were intracranially (i.c.) injected with GL261-CIITA cells into the right striatum and after 21 days challenged with parental GL261 tumor cells in the left striatum (pre-vaccinated group). After three additional weeks, animals were sacrificed, and their brains were analyzed histologically for presence and size of tumors. As a control, another group of mice were i.c. injected with GL261 cells (non-vaccinated group), and their brains were analyzed after 3 weeks as described in **Figure 1**. **(A)** Average size of GL261 tumors in pre-vaccinated and non-vaccinated mice. Bars represent mean values, and error bars indicate the SD of each group, n = 5. *p*-Values were determined *via* unpaired t-test; ***p < 0.001. **(B)** Representative histological sections of the brains harvested from pre-vaccinated (upper panels) and non-vaccinated (bottom panels) mice, at ×20 magnification; scale bar corresponds to 500 μM. Sections were stained with HE or by IHC with nestin- and synaptophysin-specific antibodies to better identify tumoral and non-tumoral tissue, respectively. Arrowheads in the HE-stained section indicate the GL261 parental tumor site (black) and the GL261-CIITA tumor site (red). HE, hematoxylin and eosin; IHC, immunohistochemistry.

Importantly, in pre-vaccinated mice, further challenge with GL261 parental tumor cells in the opposite hemisphere resulted in complete tumor rejection in 60% of cases or in highly reduced tumor growth (pre-vaccinated mice: average tumor size 0.15 mm^2^) in the remaining 40% of cases, as compared to control non-vaccinated mice (non-vaccinated mice: average tumor size 10.17 mm^2^) (unpaired Student’s t-test, *p* < 0.05) (**Figure 5A**). Brain sections of representative pre-vaccinated and non-vaccinated mice clearly depict how pre-vaccination with GL261-CIITA cells significantly impaired the growth of GL261 parental tumor in the opposite hemisphere in 40% of mice (**Figure 5B**, black arrowhead), with this effect taking the form of complete regression in the remaining 60% of mice, as compared to non-vaccinated mice.

**Figure 6:**
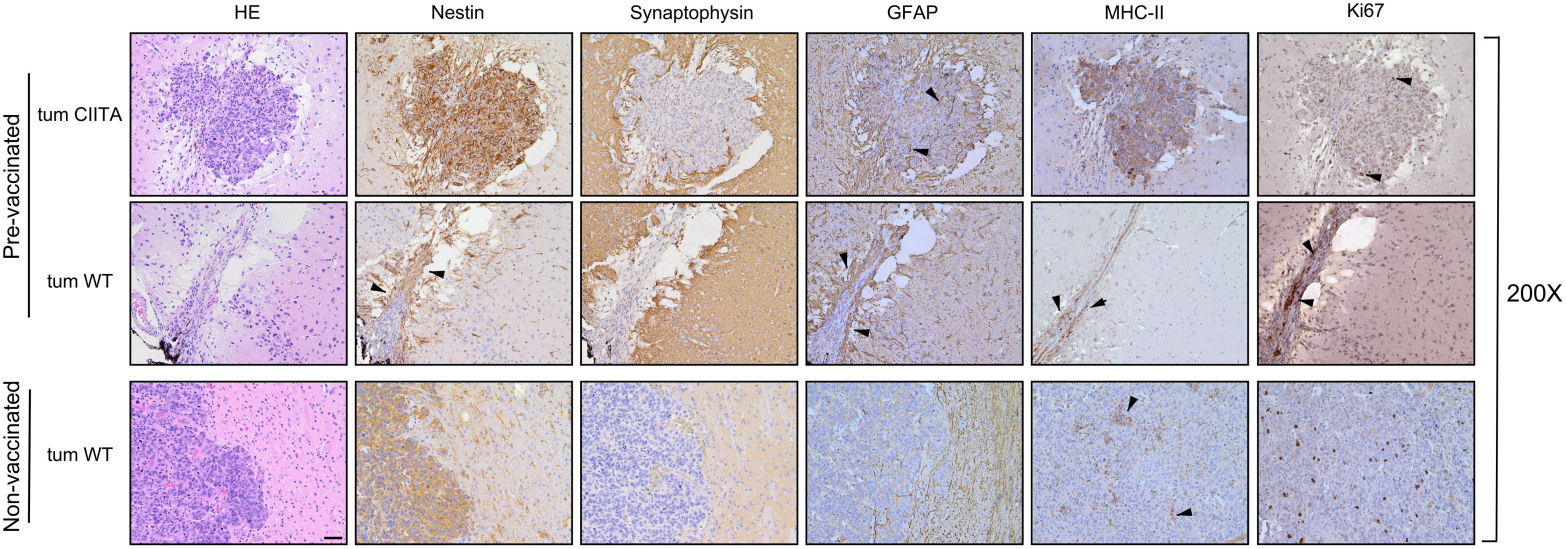
Immunohistological characterization of GL261 tumor rejection in GL261-CIITA pre-vaccinated mice. Representative histological sections of tumor tissues harvested from pre-vaccinated (upper panels, tum CIITA and tum WT) and non-vaccinated (bottom panels, tum WT) mice, at ×200 magnification; scale bar corresponds to 50 μM. HE was followed by immunohistochemical staining with nestin- and synaptophysin-specific antibodies to better identify tumoral and non-tumoral tissue, respectively. Arrowheads in the panel showing nestin staining of GL261 tumors in pre-vaccinated mice point to astrocytosis-enriched areas. MHC-II expression was specifically observed on GL261-CIITA tumor cells (pre-vaccinated, tum CIITA) and myeloid cells dispersed over the tumor bed at the site of GL261 tumor injection (pre-vaccinated, tum WT, black arrowheads) or dispersed along the GL261 tumor (non-vaccinated, tum WT, black arrowheads). Marked GFAP-positive astrocytosis was found on GL261-CIITA tumors (pre-vaccinated, tum CIITA, black arrowheads). GFAP is also markedly expressed and distributed around the tumor bed in the left striatum (pre-vaccinated, tum WT, black arrowheads). Ki67-positive cells are abundant in tumors isolated from non-vaccinated mice and decreased in GL261-CIITA tumors (pre-vaccinated, tum CIITA, black arrowheads) or markedly expressed by lymphocytes and fibroblasts over the tumor bed in the right striatum (pre-vaccinated, tum WT, black arrowheads). HE, hematoxylin and eosin; GFAP, glial fibrillary acidic protein.

Of note, in the right striatum of the GL261-CIITA-vaccinated mice, we detected only a residual tumor mass at 42 days post-injection, confirming the existence of a potent and lasting protective anti-tumor state (**Figure 5B**, red arrowhead). Importantly, although the GL261-CIITA tumors were larger at 42 days post-injection compared to the measurements taken at 21 days post-injection (unpaired Student’s t-test, *p* < 0.01), they were still notably smaller than GL261 parental tumors (unpaired Student’s t-test, *p* < 0.01) (**Supplementary Figure 7**). HE-stained sections of the GL261-injected hemisphere of pre-vaccinated mice revealed a residual tumor bed, mostly populated by fibrous cells and characterized by an edematous peripheral area and abundant inflammatory infiltrate, distributed over the entire tumor bed surface (**Figure 6**, pre-vaccinated, tum wild type (WT)). These features are consistent with a complete regression of the challenged GL261 tumor. Synaptophysin staining clearly defined the boundaries of the tumor bed (**Figure 6**, pre-vaccinated, tum WT).

Consistent with the results at 21 days post tumor cell injection, nestin was strongly expressed on both GL261-CIITA tumor cells and GL261 parental tumor and in peritumoral astrocytes in pre-vaccinated and non-vaccinated mice, respectively (**Figures 5**, **6**; compare pre-vaccinated, tum CIITA, to non-vaccinated, tum WT). Interestingly, we also found a marked and dense nestin-positive peritumoral astrocytic cell population, concentrated along the tumor bed of challenged GL261 tumor in pre-vaccinated mice (**Figure 6**; pre-vaccinated, tum WT, black arrowheads).

Consistent with what we observed at 21 days post tumor implantation, in pre-vaccinated mice, GFAP staining revealed intense astrocytosis in GL261-CIITA tumors, forming a complex matrix surrounding the tumor mass, and also partially infiltrating the tumor (**Figure 6**; pre-vaccinated, tum CIITA, black arrowheads). Notably, in the opposite hemisphere, GFAP was markedly expressed and distributed around the tumor bed (**Figure 6**; pre-vaccinated, tum WT, black arrowheads). Conversely, in non-vaccinated mice, the astrocytic population was less abundant and was not completely organized to form a peritumoral lining (**Figure 6**; non-vaccinated, tum WT).

Cellular proliferation rate, as evaluated by Ki67 staining, clearly indicated a high level of proliferation in GL261 parental tumors in non-vaccinated mice’, and very low proliferation in GL261-CIITA tumors also at 42 days post-injection (**Figure 6**; pre-vaccinated, tum CIITA, black arrowheads). Importantly, in pre-vaccinated mice, Ki67 staining revealed a significant increase in lymphocyte and fibroblast proliferation rates over the tumor bed (**Figure 6**; pre-vaccinated, tum WT, black arrowheads).

Furthermore, we evaluated MHC-II expression in both pre-vaccinated and non-vaccinated mice, confirming its expression by GL261-CIITA tumor cells (**Figure 6**; pre-vaccinated, tum CIITA). Interestingly, in pre-vaccinated mice, we found that MHC-II-positive myeloid cells, most likely macrophages, were dispersed over the tumor bed at the site of GL261 tumor injection (**Figure 6**; pre-vaccinated, tum WT, black arrowheads). Conversely, this population was only very marginally observable in tumors of non-vaccinated mice, dispersed along the GL261 tumor (**Figure 6**; non-vaccinated, tum WT, black arrowheads).

### Preventive GL261-CIITA vaccination results in a dramatic increase in infiltrating T lymphocytes in GL261 tumors

**Figure 7:**
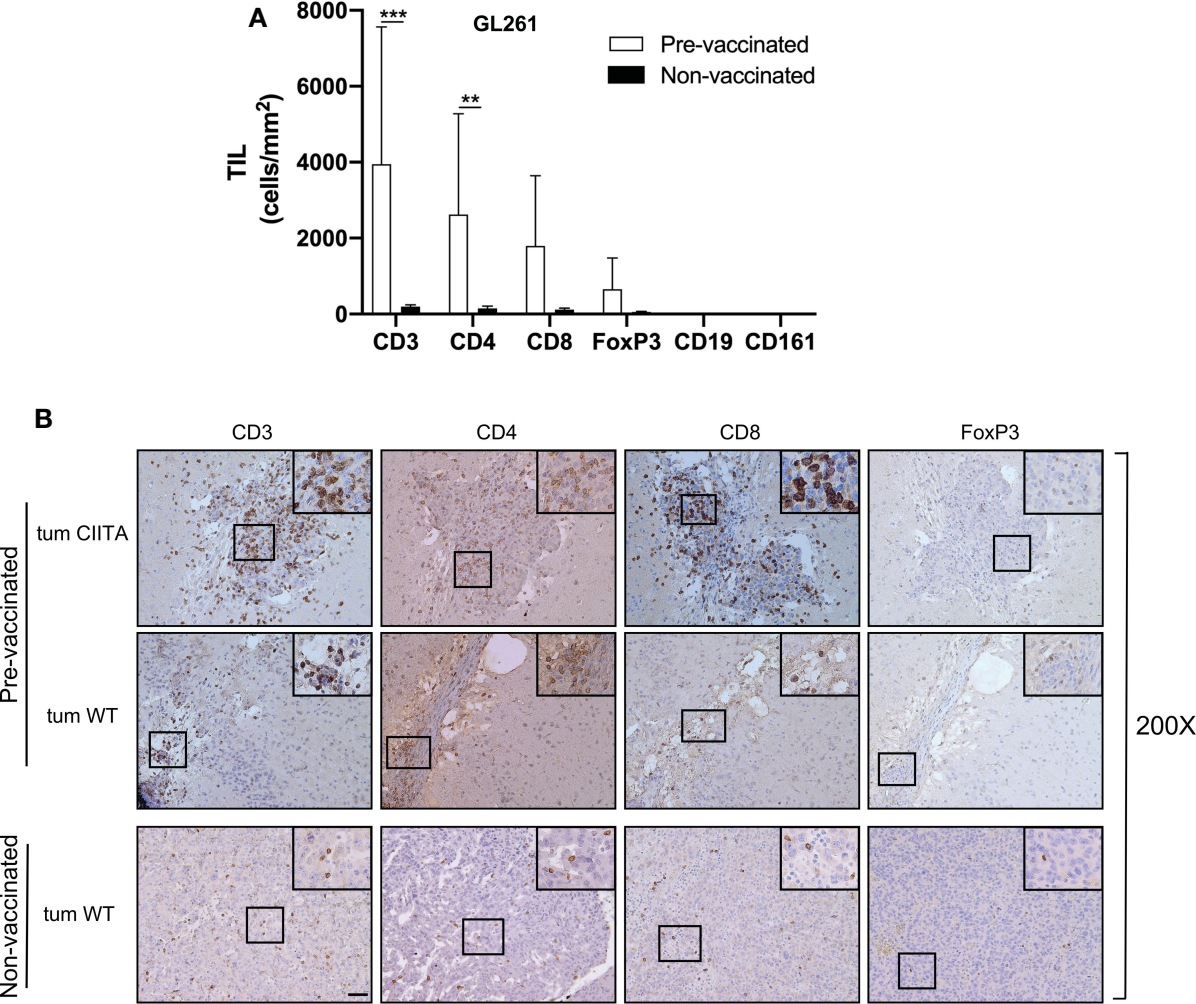
Rejected GL261 parental tumors in pre-vaccinated mice are strongly infiltrated by CD4 T cells. **(A)** Bars represent the average number of listed tumor-infiltrating lymphocytes (TILs) in the GL261 parental tumor site of GL261-CIITA pre-vaccinated or non-vaccinated mice. Bars represent mean values, and error bars indicate the SD within each group, n = 5. *p*-Values were determined *via* unpaired t-test; CD3: ***p < 0.001; *CD4*: **p < 0.01; the average number of both CD8 and FoxP3 did not differ significantly between the two groups. CD19 and CD161 were not significantly represented. **(B)** Representative immunohistology images of tumor sections as described in **(A)** The first series of horizontal panels depict GL261-CIITA tumors in pre-vaccinated mice at 42 days after inoculum (pre-vaccinated, tum CIITA), stained for the specific markers listed at the top. The second series of panels depict GL261 parental tumors in GL261-CIITA pre-vaccinated mice (pre-vaccinated, tum WT). The final series of horizontal panels depict GL261 parental tumors in non-vaccinated mice (non-vaccinated, tum WT). Small square boxes are the areas represented in the corresponding large square boxes of each IHC image. Images were taken at ×200 magnification; scale bar corresponds to 50 μM. Large square boxes were taken at ×400 magnification. Note that selected areas in IHC images of GL261 parental tumors of non-vaccinated mice are taken in the rare zones in which positive cells for the selective marker were present. IHC, immunohistochemistry.

To further investigate the immunological correlates of tumor rejection in pre-vaccinated mice challenged with GL261 parental tumor cells, IHC analysis was performed using anti-CD3, anti-CD4, anti-CD8, and anti-FoxP3 antibodies. Quantification of tumor-infiltrating cells was performed histologically as described in the Materials and Methods section. The number of tumor-infiltrating T cells was significantly increased in GL261 tumors in pre-vaccinated mice as compared to the number occurring in GL261 tumors in non-vaccinated mice (unpaired Student’s t-test, CD3+: *p* < 0.0001; CD4+: *p* < 0.001) (**Figure 7A**). Tumor infiltrate was mainly characterized by CD4+ T cells, and the average number of CD4+ T cells was 16-fold higher in GL261 tumors in pre-vaccinated mice as compared to GL261 tumors in non-vaccinated mice (CD4+: 2,622 cells/mm^2^ in challenged GL261; CD4+: 153 cells/mm^2^ in GL261). We also observed an increase in CD8+ T cells, although this was not statistically significant compared to non-vaccinated mice (CD8+: 1,794 cells/mm^2^ in challenged GL261; CD8+: 114 cells/mm^2^ in GL261). FoxP3+ T cells were less abundant compared to both CD4+ and CD8+ T cells, and we found no statistically significant difference between pre-vaccinated and non-vaccinated mice (**Figure 7A**). As observed in tumor tissues originating from GL261 parental cell injection, CD19+ B cells and CD161+ NK cells were not detected (**Figure 7A**). Notably, microscopic evaluation of immunostained sections showed that in challenged GL261 brain hemispheres, CD4+ T cells were widely distributed within the tumor bed and along its margins (**Figure 7B**; pre-vaccinated, tum WT, insets ×400), whereas they were less abundant and dispersed throughout the GL261 tumor mass in non-vaccinated mice (**Figure 7B**; non-vaccinated, tum WT, insets ×400). As observed on day 21 in GL261-CIITA-injected mice, at 42 days after injection of the same cells, the residual tumors were found to be strongly infiltrated by CD4+ and CD8+ T cells with a peculiar cluster formation inside the neoplastic area (**Figure 7B**; pre-vaccinated, tum CIITA, insets ×400), and with no significant difference in number in comparison to day 21 (**Supplementary Figure 8**).

## Discussion

The described investigation was undertaken to find alternative ways to overcome the present dismal outcomes of therapeutic options for GBM. Radiotherapy, chemotherapy, and recent immunotherapy approaches to GBM are still insufficient to provide a decent life expectancy for GBM patients. Our main aim was to assess, in an experimental animal model, whether our approach of vaccination with CIITA-induced, MHC-II-expressing tumor cells is suitable for application in cases of GBM, a tumor that shows distinct idiosyncrasies with respect to other neoplasias for its extreme malignancy and for its specific localization in the CNS, an organ that is relatively protected from immune attack because of the BBB. We were motivated in this direction by previous success obtained with diverse experimental mouse tumor models, both carcinomas and sarcomas, of distinct genetic backgrounds (reviewed in (24, 43, 44)), and by recent discoveries, not only of a lymphatic system in mice along the draining cerebral sinuses (7, 8) but also of the presence of a local source of functional immune cells resident in the bone marrow of the skull that can be mobilized into the brain (9). The results obtained are unprecedented in several ways and partially justify our present excitement. Indeed, highly tumorigenic GL261 mouse GBM cells become strongly immunogenic and are rejected or their growth greatly reduced when they are transduced with CIITA and injected orthotopically into the mouse brain. The immunogenic nature of the rejection/retardation of tumor growth of GL261-CIITA cells was demonstrated by the fact that GL261-CIITA tumors were rapidly infiltrated by CD4+ and CD8+ T cells, but not B and NK cells, and, most importantly, by the acquisition of a protective memory response to subsequent challenge in the opposite hemisphere with parental GL261. Specific CD4+ and CD8+ T-cell infiltration was very marginally observed in the brains of control mice injected with GL261 parental tumor cells but was observed to a significant extent in GL261 tumors of GL261-challenged mice after GL261-CIITA vaccination. These results hold, in our opinion, further relevance because they indicate that immune cells generated by stimulation of GL261-CIITA tumor cells can travel across the brain and reach specific targets outside the original site of recognition.

Based on these and previous results of our group (23, 26, 27) that have demonstrated, both by cell depletion and by adoptive cell transfer, the anti-tumor effect of primed CD4+ Th cells, we believe that these cells are instrumental in triggering and activating tumor-specific naïve CD8+ T cells to make them fully mature anti-tumor CTL effectors. Nevertheless, we cannot entirely exclude, as a parallel mechanism, the direct action of tumor-specific CD4+ Th cells as cytotoxic effectors, as shown in several animal models (45, 46) and more recently in human tumors by single-cell sequencing (47, 48).

Furthermore, our results strongly suggest that CIITA-induced MHC class II cell surface molecules in GL261-CIITA tumor cells are the key element in triggering the adaptive CD4+ Th cell anti-tumor response, most likely serving as antigen-presenting molecules for tumor-specific GBM peptide antigens. This implies that surrogate antigen-presenting cells play a role *in vivo* for GL261-CIITA tumor cells, as previously demonstrated in other tumor models (23) and anticipated previously by *in vitro* studies (29, 49). Within this framework, it is important to underline the significance of the kinetics of tumor growth over time (**Figure 4**), which clearly demonstrated that GL261-CIITA tumor cells were arrested in their proliferation very early after the inoculum, strongly suggesting that their recognition by the immune system was a rapid event *in vivo*, compatible with their direct surrogate antigen-presenting function with respect to tumor-specific Th cells. Future studies will be directed toward clarification of the MHC-II-bound tumor-associated peptides that constitute the repertoire of GBM-specific antigens expressed *de novo* on the cell surface of GL261-CIITA tumor cells. This is a crucial element for the construction of a future vaccine against GBM, particularly because we have already identified many new and potential GBM-specific tumor antigens in our recent studies on the characterization of the MHC-II immunopeptidome of human GBM cells transduced with CIITA (28). The animal model described in this article will be then ideal for the evaluation *in vivo* of the specificity and the efficiency of the adaptive immune response against well-selected GBM-specific, MHC-II-bound tumor peptides.

Indeed, human GBM cells that stably express CIITA are decorated with remarkably high levels of HLA-II–peptide complexes (28). This renders these cells very attractive for antigen discovery endeavors and thus, as outlined above, for the isolation of tumor-specific peptides to be used for the construction of novel vaccines amenable to clinical application. Moreover, viral vectors containing expressible CIITA, alone or in association with oncolytic viruses, or agents that can overcome the blood–brain barrier, could be used to target established GBM tumors and make them more immunogenic in order to trigger and/or increase the stimulation of tumor-specific Th cells (50).

Additional findings of the present investigation deserve attention and preliminary comments. One of these findings was the substantial increase in infiltrating FoxP3+ T cells in GL261-CIITA tumors with respect to GL261 parental tumors, which resulted in a decreased ratio of the putative CD8+ T cell effectors to FoxP3+ T cells in the latter tumors, despite the fact that a clear 13.7-fold increase in CD8+ T cells was observed in GL261-CIITA tumors compared to GL261 parental tumors. FoxP3+ T cells are usually associated with a negative regulatory function (Treg) over CD4+ Th cells, and their presence in the tumor microenvironment often indicates an immunosuppressive function toward anti-tumor T cells (42). Moreover, an increase in CD8+/Treg+ T cells is usually associated with a better response to certain immunotherapies (51). The fact that, in our study, they were present mostly in tumors undergoing rejection or strong retardation of growth suggests that their recruitment was not associated with an inhibitory function toward the anti-tumor response or that they were inhibited in their suppressive function by a mechanism that remains unclear, but would certainly be related to the immune response triggered by GL261-CIITA tumor cells. The kinetics of recruitment and accurate functional analysis of these FoxP3+ T cells certainly deserve attention in future studies. Another interesting finding of our study was the relevant reactive astrocytosis observed in GL261-CIITA tumor tissue as compared to GL261 parental tumor tissue. In response to a variety of brain injuries, including BBB damage and cancer, astrocytes undergo a process of reactive gliosis that involves upregulation of the intermediate filament GFAP, as well as a number of growth factors, inflammatory cytokines, and extracellular matrix proteins (52). In brain tumors, this process can be associated with facilitation or protection of tumor growth (53). Thus, it was relatively unexpected that we would find higher activation of astrocytes in GL261-CIITA tumors that indeed are undergoing tumor arrest and rejection by the immune system as compared to the aggressive behavior of GL261 parental tumors. A possible explanation would be that reactive astrocytosis is indeed a component of the inflammatory response that accompanies the tumor state, and, as such, activated astrocytes contribute to the formation of a functional barrier, often designated a “glial scar”, to restrict inflammation and restore the BBB (54).

In conclusion, we have provided evidence that glioblastoma, often seen as scarcely immunogenic because of their reduced mutational burden and their intrinsic immunosuppressive, “cold” microenvironment (55, 56), could be rendered highly immunogenic and prone to rejection by the host, at least in the experimental animal model described, by forcing the expression of the MHC class II transactivator CIITA and thus its recognition by the immune system. A combination of *in vivo* induction of CIITA and characterization of the MHC-II-bound immunopeptidome may be, in the near future, a possible novel approach to treatment of this still therapeutically unresponsive and deadly form of tumor.

## Data availability statement

The original contributions presented in the study are included in the article/**Supplementary Material**. Further inquiries can be directed to the corresponding authors.

## Ethics statement

The animal study was reviewed and approved by the University of Insubria Internal Ethical Committee CESA (Project 05–2020) and by the Italian Ministry of Health (Authorization number: 812-2020-PR).

## Author contributions

Conceptualization: FC and RA. Data collection: FC, AG, MS, AC, MC, RA, and GF. Investigation: FC, AG, MS, AC, MC, and GF. Supervision: AC, MC, RA, and GF. Funding acquisition: GF. Writing of original draft: RA and GF. Writing, review and editing: FC, AG, MS, AC, MC, FS, RA, and GF. All authors contributed to the article and approved the submitted version.

## Supplementary material

Supplementary Figure 1 Histological parameters used to measure tumor size and to count the cells infiltrating the tumor. The tumor size and the immune infiltrate have been evaluated as specified in Material and Methods. Peritumoral tissue (PT), invasive margin (IM) and central tumor (CT) are indicated (200x magnification). The boxed image was enlarged at 400x magnification.

Supplementary Figure 2 Expression of MHC-I and MHC-II cell surface molecules in GL261 glioma cells after stable expression of CIITA. (A) The stable expression of CIITA in GL261 GBM cells induces MHC-II expression (GL261-CIITA). MHC-I and MHC-II cell surface expression was assessed by immunofluorescence and FACS analysis. Histograms represent fluorescence profiles of the cells indicated on the left incubated with specific anti-MHC-I or MHC-II mAbs (solid line). Controls (dashed line) are cells incubated with the specific isotype control. Mean fluorescence (m.f.) values are expressed in the abscissa as arbitrary units (a.u.). A representative experiment out of three independent experiments is shown. (B) Cell lysates obtained from either GL261 or GL261-CIITA cells (4X10^6^ cells) were analyzed for the presence of CIITA by western blotting. As a control for loading, the expression of α-tubulin was also evaluated. Molecular sizes in kilodaltons are shown in the left of the figure.

Supplementary Figure 3 Ki67 positive cells are significantly fewer in GL261-CIITA than in GL261 parental tumors. C57BL/6 mice received intracranial injection of 3x10^4^ GL261 or GL261-CIITA glioma cells. Day-21 tumors were removed from mice and brain tumor section stained for immunohistochemistry. Slides from the brain tissues isolated from GL261 or GL261-CIITA tumor bearing mice were subjected to immunohistochemical staining with anti-Ki67 or anti-GFAP antibodies. Representative images with high magnification (200x) are shown. Scale bar corresponds to 50 μM. Black arrowheads indicate specific cell staining as specified in the text.

Supplementary Figure 4 The expression of CIITA in GL261 GBM cells does not affect their proliferation rate *in vitro*. Bar graphs represent the number of GL261 and GL261-CIITA cells counted at the specific time points listed in the abscissa, as assessed by trypan blue exclusion assay. Bars represent the mean ± S.D. from three independent experiments.

Supplementary Figure 5 Distribution of distinct T cell subpopulations in the various districts of the tumor tissue. Bar graphs represent the average number of CD3, CD4, CD8 and FoxP3, tumor infiltrating lymphocytes (TIL) counted from histopathological sections in the three different regions indicated in **Supplementary Figure 1**: central tumor (CT, upper left graph), tumor invasive margine (IM, upper right graph) and peritumoral region (PT, bottom graph). Data are represented as mean values and error bars indicate the SD for each group. n=6, P-values were determined using unpaired t test; *****P<*0.0001; ****p<*0.001; ***p<*0.01; **p<*0.05.

Supplementary Figure 6 Challenged GL261 tumors are not infiltrated by CD19+, CD161+ or CD11c+ cells. C57BL/6 mice received intracranial injection of 3x10^4^ GL261 or GL261-CIITA glioma cells. Day-21 tumors were removed from mice and brain tumor sections stained for immunohistochemistry. Slides from the brain tissues isolated from GL261 or GL261-CIITA tumor bearing mice were subjected to immunohistochemical staining with anti-CD19, anti-CD161, anti-CD11b and anti-CD11c antibodies. Small square boxes are the areas represented in the corresponding large square boxes of each single IHC image. Images are taken at 200x magnification. Scale bar corresponds to 50 μM. Large square boxes are taken at 400x magnification.

Supplementary Figure 7 Size of GL261-CIITA tumors, when present, at 42 days is dramatically reduced as compared to the size of parental GL261 parental tumors at 21 days. Average tumor size of GL261-CIITA at 21 (n=10), and 42 (n=5) days post cell inoculum, and of GL261 parental tumor at 21 (n=7) days post cell inoculum. Data are represented as mean values and error bars indicate the SD of each group. P-values were determined using unpaired t test; GL261-CIITA, day 21 *versus* day 42, ***p*<0.01; GL261-CIITA day 42 versus GL261 day 21, ***p*<0.01.

Supplementary Figure 8 GL261-CIITA tumors are highly infiltrated by T cells up to 42 days after tumor cells injection. C57BL/6 mice received intracranial injection of 3x10^4^ GL261 or GL261-CIITA glioma cells. At day 21 from injection, mice were sacrificed, brains were removed, and serial sections of the brain were carried out to be stained with anti-CD3, anti-CD4, anti-CD8, anti-FoxP3, anti-CD19, and anti-CD161 antibodies. Bar graphs represent the average number of CD3, CD4, CD8, FoxP3, CD19 and CD161 tumor infiltrating lymphocytes (TIL) measured from histopathological sections of GL261-CIITA at 21 (n=7) day or 42 (n=5) days post-injection. Data are represented as mean values and error bars indicate the SD of each group.
