# Supplementary figures and images for: Protective anti-tumor vaccination against glioblastoma expressing the MHC class II transactivator CIITA
Source: Front Immunol. 2023 Mar 13;14:1133177. doi: 10.3389/fimmu.2023.1133177 (PMC10040613; doi:10.3389/fimmu.2023.1133177)

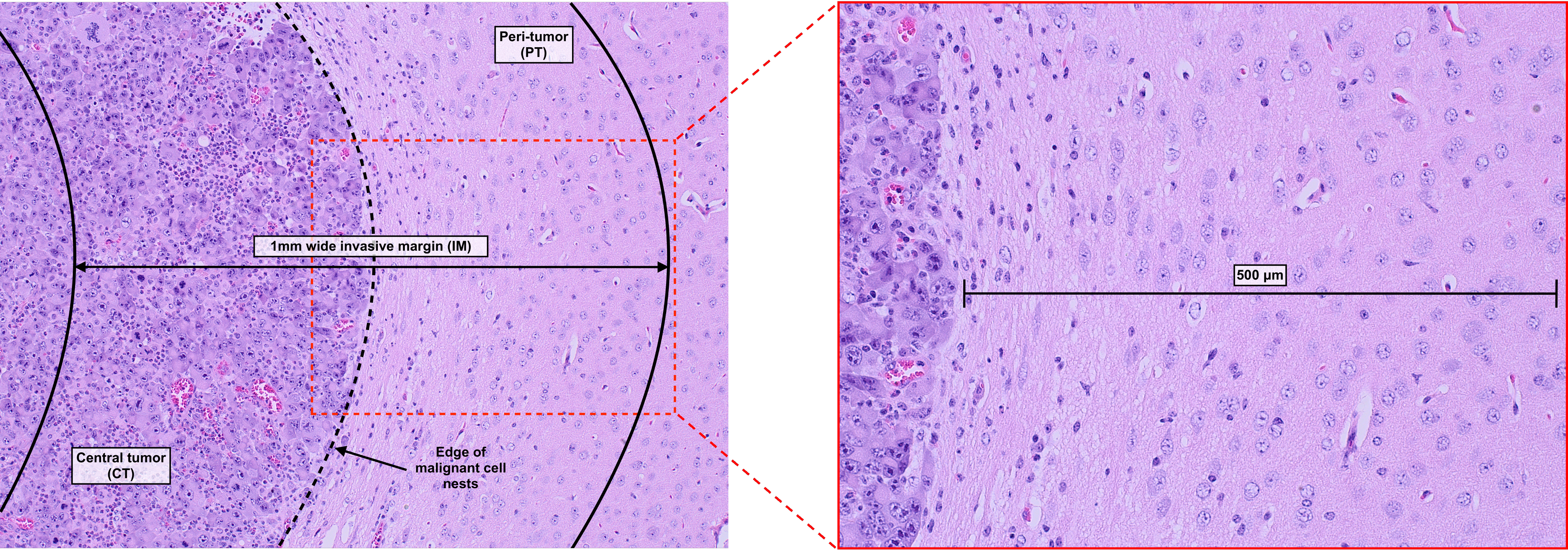

Supplement: Supplementary Figure 1 — Histological parameters used to measure tumor size and to count the cells infiltrating the tumor. The tumor size and the immune infiltrate have been evaluated as specified in Material and Methods. Peritumoral tissue (PT), invasive margin (IM) and central tumor (CT) are indicated (200x magnification). The boxed image was enlarged at 400x magnification. [file Image_1.tiff]

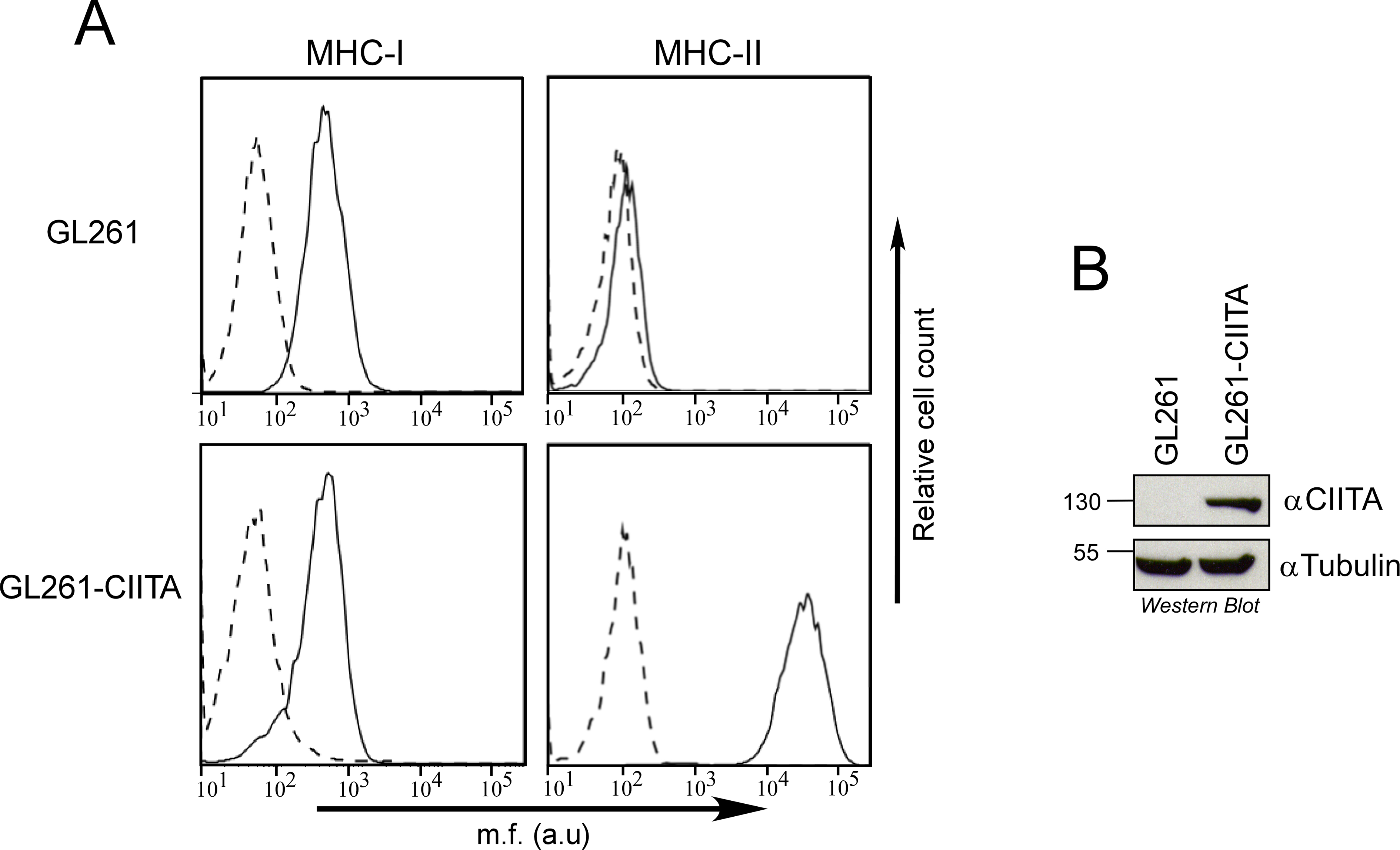

Supplement: Supplementary Figure 2 — Expression of MHC-I and MHC-II cell surface molecules in GL261 glioma cells after stable expression of CIITA. (A) The stable expression of CIITA in GL261 GBM cells induces MHC-II expression (GL261-CIITA). MHC-I and MHC-II cell surface expression was assessed by immunofluorescence and FACS analysis. Histograms represent fluorescence profiles of the cells indicated on the left incubated with specific anti-MHC-I or MHC-II mAbs (solid line). Controls (dashed line) are cells incubated with the specific isotype control. Mean fluorescence (m.f.) values are expressed in the abscissa as arbitrary units (a.u.). A representative experiment out of three independent experiments is shown. (B) Cell lysates obtained from either GL261 or GL261-CIITA cells (4X106 cells) were analyzed for the presence of CIITA by western blotting. As a control for loading, the expression of α-tubulin was also evaluated. Molecular sizes in kilodaltons are shown in the left of the figure. [file Image_2.jpeg]

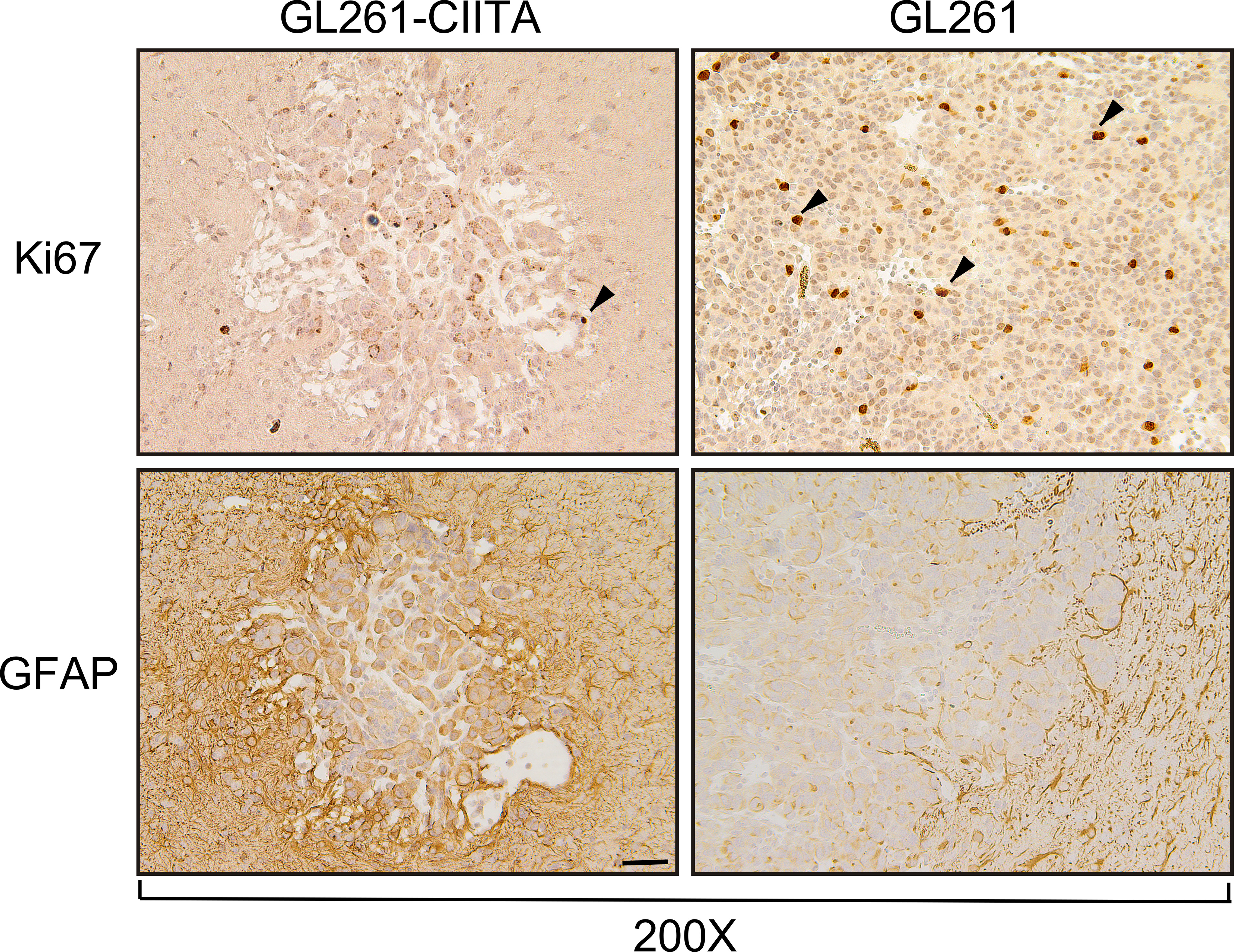

Supplement: Supplementary Figure 3 — Ki67 positive cells are significantly fewer in GL261-CIITA than in GL261 parental tumors. C57BL/6 mice received intracranial injection of 3x10^4^ GL261 or GL261-CIITA glioma cells. Day-21 tumors were removed from mice and brain tumor section stained for immunohistochemistry. Slides from the brain tissues isolated from GL261 or GL261-CIITA tumor bearing mice were subjected to immunohistochemical staining with anti-Ki67 or anti-GFAP antibodies. Representative images with high magnification (200x) are shown. Scale bar corresponds to 50 μM. Black arrowheads indicate specific cell staining as specified in the text. [file Image_3.jpeg]

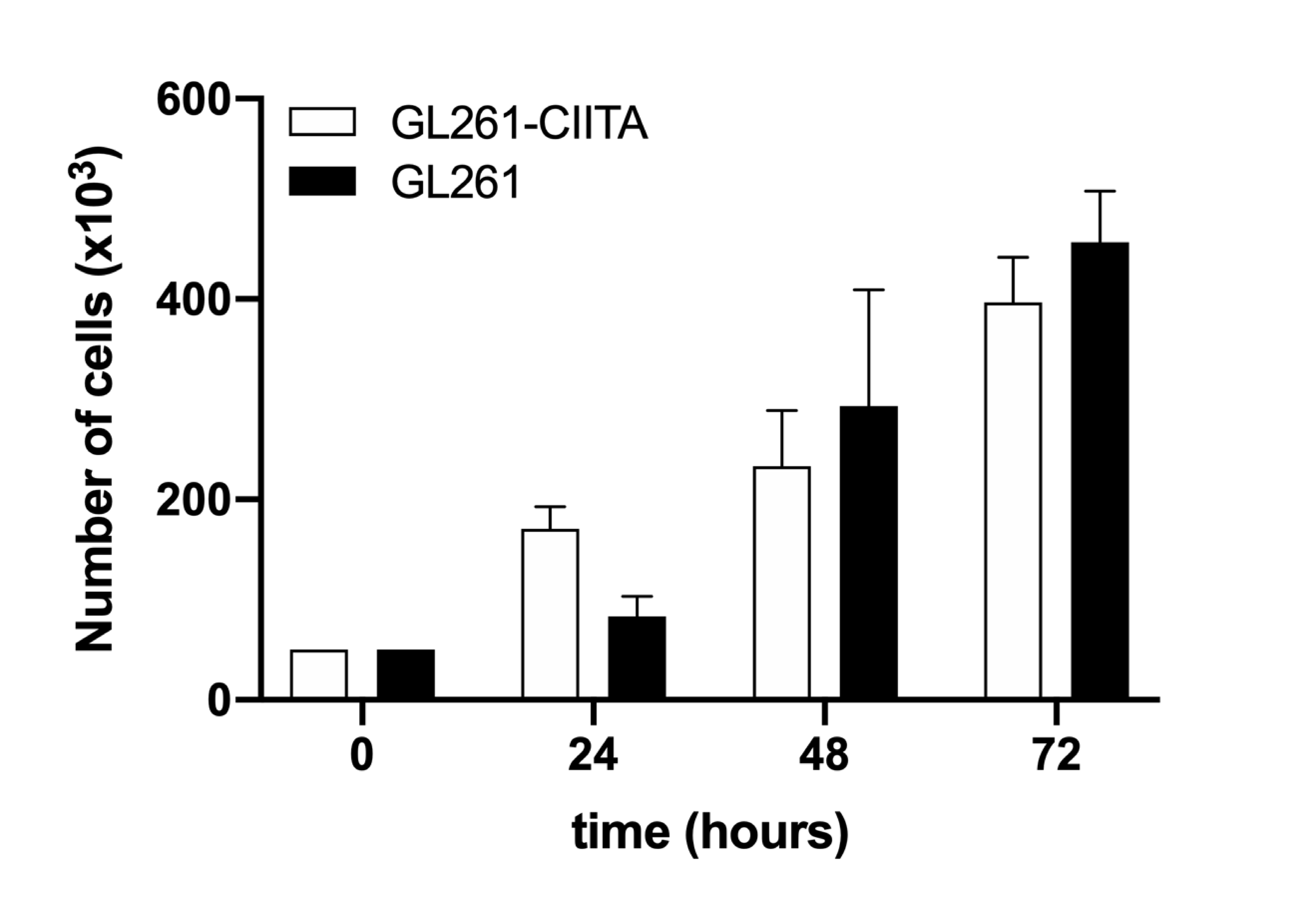

Supplement: Supplementary Figure 4 — The expression of CIITA in GL261 GBM cells does not affect their proliferation rate in vitro. Bar graphs represent the number of GL261 and GL261-CIITA cells counted at the specific time points listed in the abscissa, as assessed by trypan blue exclusion assay. Bars represent the mean ± S.D. from three independent experiments. [file Image_4.jpeg]

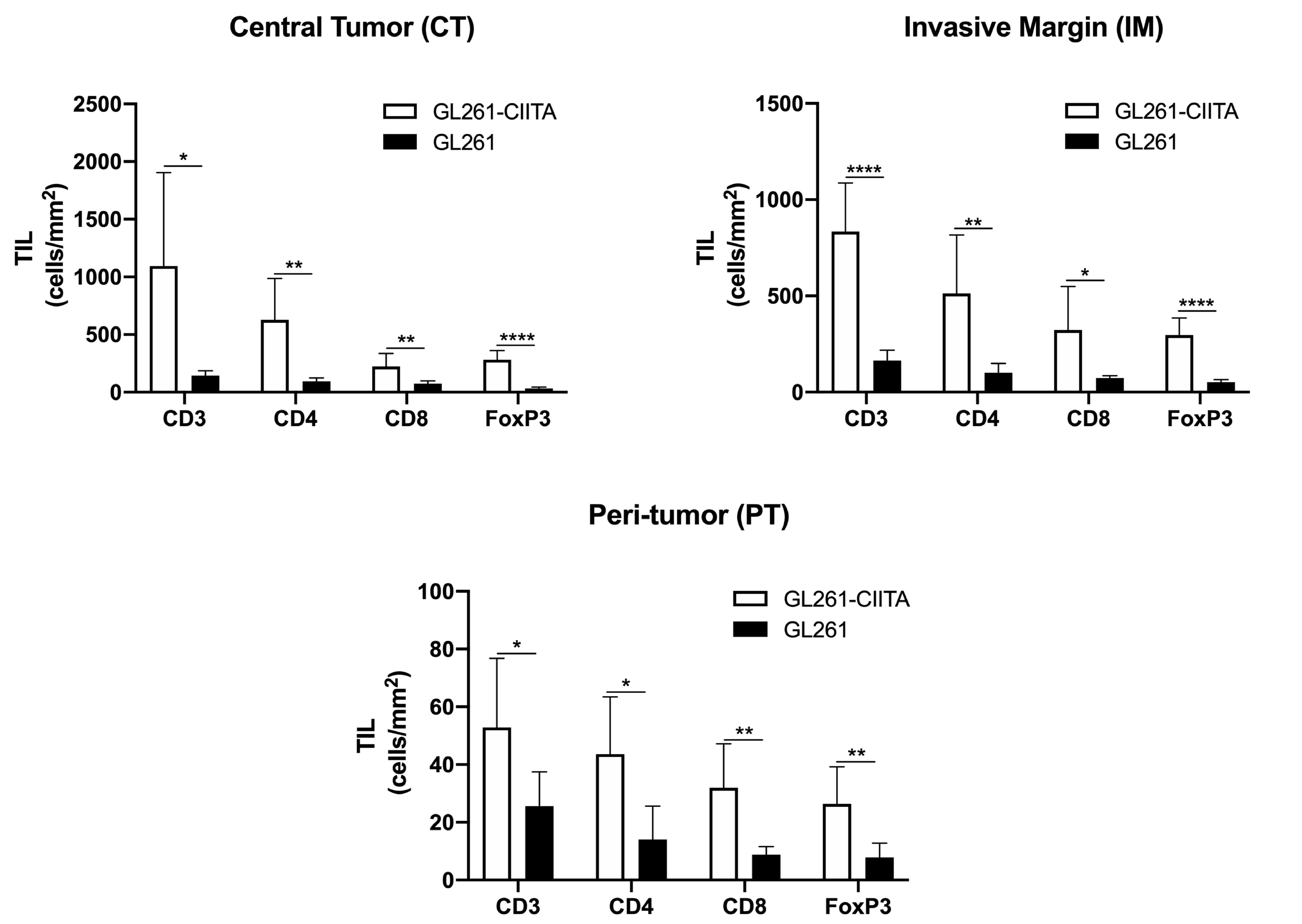

Supplement: Supplementary Figure 5 — Distribution of distinct T cell subpopulations in the various districts of the tumor tissue. Bar graphs represent the average number of CD3, CD4, CD8 and FoxP3, tumor infiltrating lymphocytes (TIL) counted from histopathological sections in the three different regions indicated in Supplementary Figure 1 : central tumor (CT, upper left graph), tumor invasive margine (IM, upper right graph) and peritumoral region (PT, bottom graph). Data are represented as mean values and error bars indicate the SD for each group. n=6, P-values were determined using unpaired t test; ****P<0.0001; ***p<0.001; **p<0.01; *p<0.05. [file Image_5.jpeg]

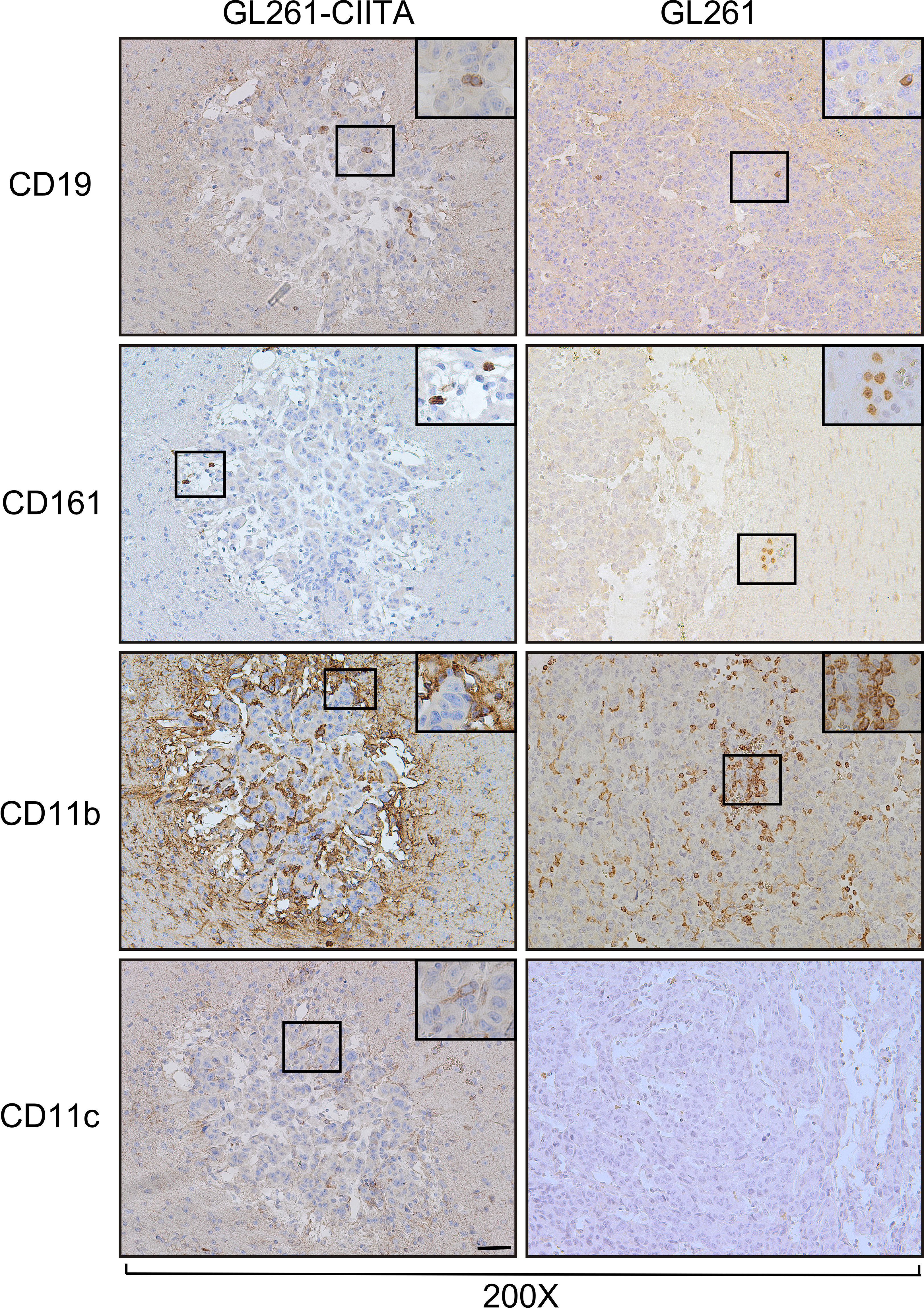

Supplement: Supplementary Figure 6 — Challenged GL261 tumors are not infiltrated by CD19+, CD161+ or CD11c+ cells. C57BL/6 mice received intracranial injection of 3x10^4^ GL261 or GL261-CIITA glioma cells. Day-21 tumors were removed from mice and brain tumor sections stained for immunohistochemistry. Slides from the brain tissues isolated from GL261 or GL261-CIITA tumor bearing mice were subjected to immunohistochemical staining with anti-CD19, anti-CD161, anti-CD11b and anti-CD11c antibodies. Small square boxes are the areas represented in the corresponding large square boxes of each single IHC image. Images are taken at 200x magnification. Scale bar corresponds to 50 μM. Large square boxes are taken at 400x magnification. [file Image_6.jpeg]

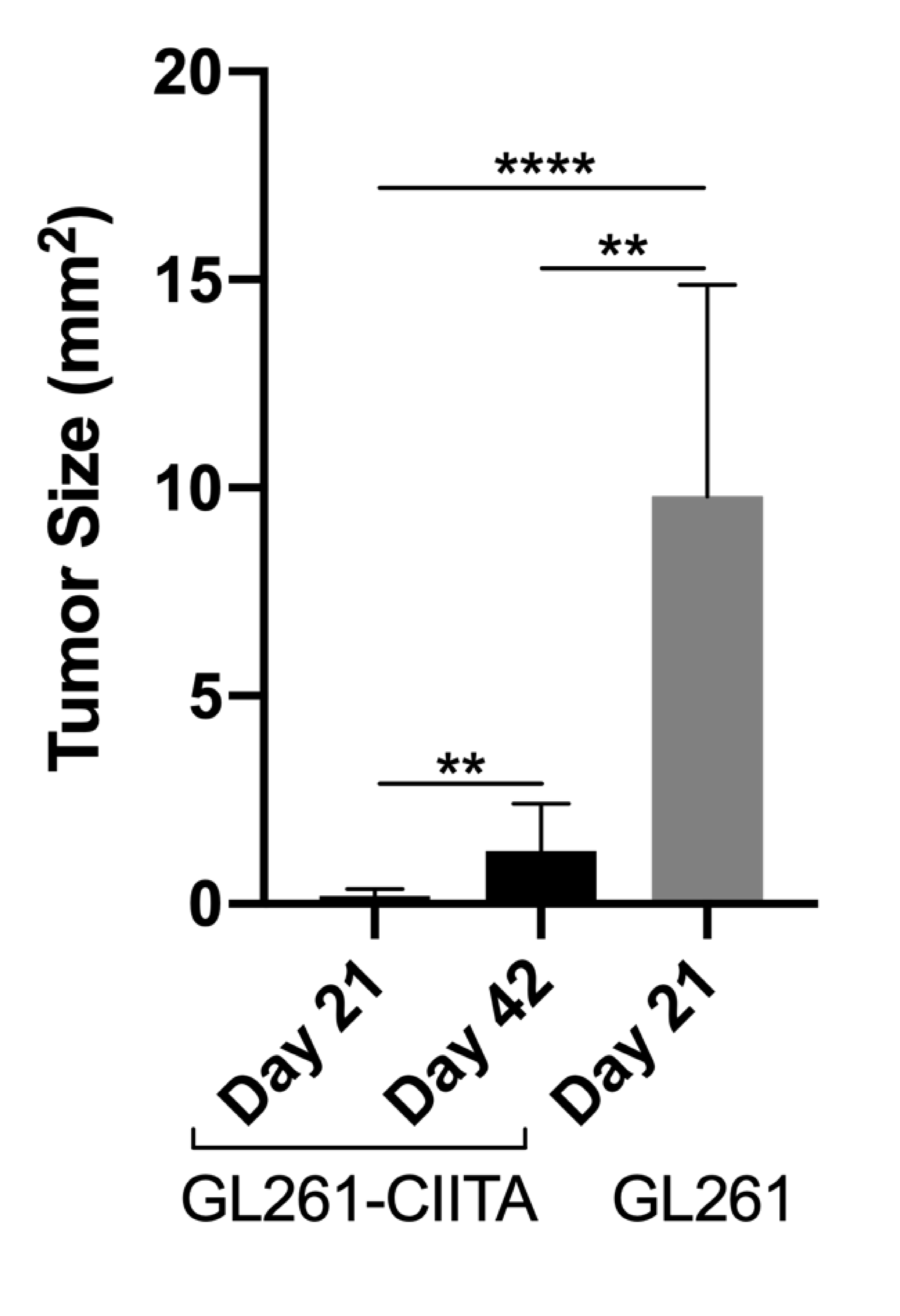

Supplement: Supplementary Figure 7 — Size of GL261-CIITA tumors, when present, at 42 days is dramatically reduced as compared to the size of parental GL261 parental tumors at 21 days. Average tumor size of GL261-CIITA at 21 (n=10), and 42 (n=5) days post cell inoculum, and of GL261 parental tumor at 21 (n=7) days post cell inoculum. Data are represented as mean values and error bars indicate the SD of each group. P-values were determined using unpaired t test; GL261-CIITA, day 21 versus day 42, **p<0.01; GL261-CIITA day 42 versus GL261 day 21, **p<0.01. [file Image_7.tif]

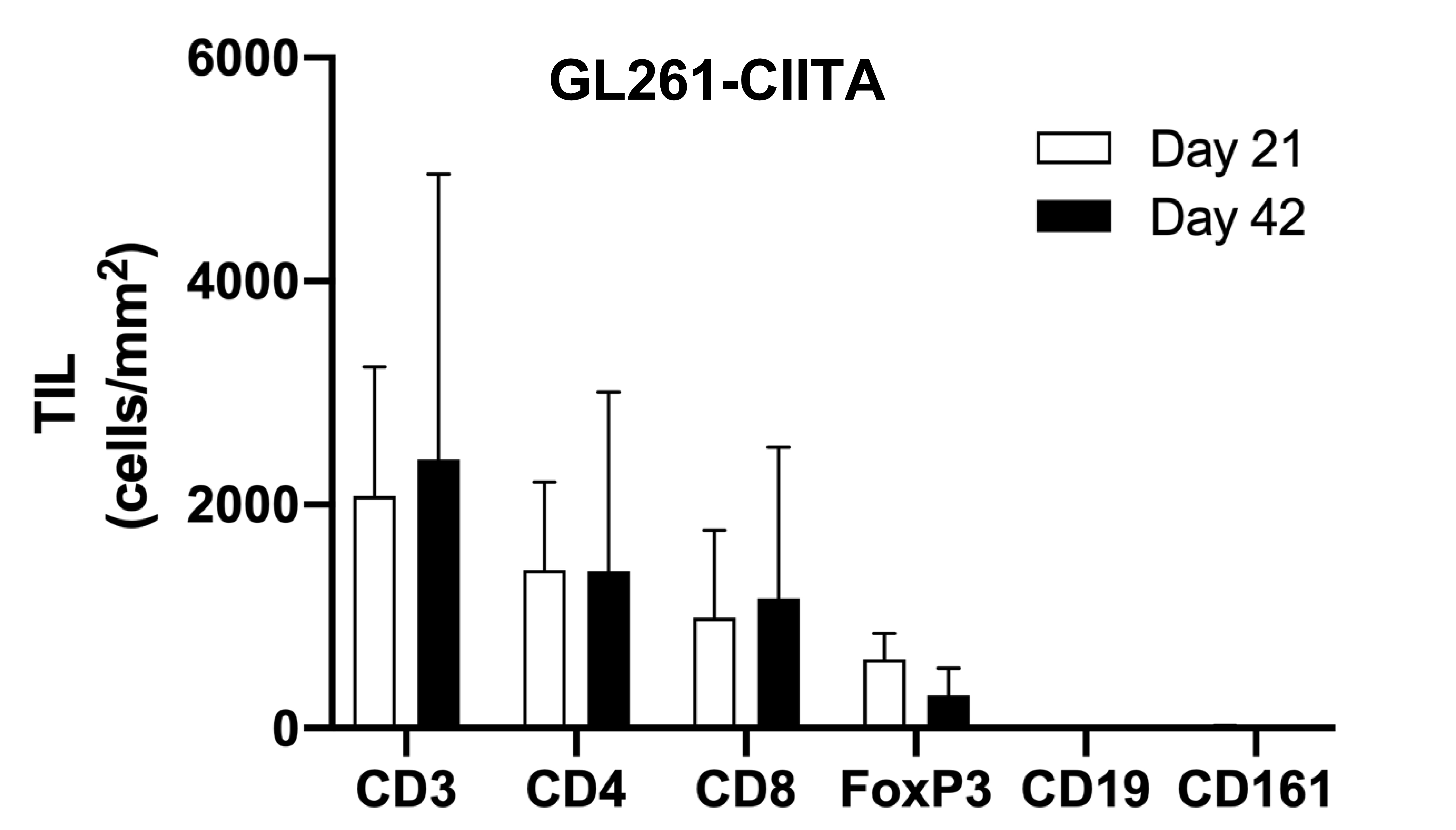

Supplement: Supplementary Figure 8 — GL261-CIITA tumors are highly infiltrated by T cells up to 42 days after tumor cells injection. C57BL/6 mice received intracranial injection of 3x10^4^ GL261 or GL261-CIITA glioma cells. At day 21 from injection, mice were sacrificed, brains were removed, and serial sections of the brain were carried out to be stained with anti-CD3, anti-CD4, anti-CD8, anti-FoxP3, anti-CD19, and anti-CD161 antibodies. Bar graphs represent the average number of CD3, CD4, CD8, FoxP3, CD19 and CD161 tumor infiltrating lymphocytes (TIL) measured from histopathological sections of GL261-CIITA at 21 (n=7) day or 42 (n=5) days post-injection. Data are represented as mean values and error bars indicate the SD of each group. [file Image_8.jpeg]
